# A Comprehensive Review of the ^1^H-MRS Metabolite Spectrum in Autism Spectrum Disorder

**DOI:** 10.3389/fnmol.2016.00014

**Published:** 2016-03-09

**Authors:** Talitha C. Ford, David P. Crewther

**Affiliations:** Faculty of Health, Arts and Design, Centre for Human Psychopharmacology, Swinburne University of TechnologyMelbourne, VIC, Australia

**Keywords:** autism spectrum disorder, ^1^H-MRS, brain metabolites, phenotype correlates, review

## Abstract

Neuroimaging studies of neuropsychiatric behavior biomarkers across spectrum disorders are typically based on diagnosis, thus failing to account for the heterogeneity of multi-dimensional spectrum disorders such as autism (ASD). Control group trait phenotypes are also seldom reported. Proton magnetic resonance spectroscopy (^1^H-MRS) measures the abundance of neurochemicals such as neurotransmitters and metabolites and hence can probe disorder phenotypes at clinical and sub-clinical levels. This detailed review summarizes and critiques the current ^1^H-MRS research in ASD. The literature reports reduced N-acetylaspartate (NAA), glutamate and glutamine (Glx), γ-aminobutyric acid (GABA), creatine and choline, and increased glutamate for children with ASD. Adult studies are few and results are inconclusive. Overall, the literature has several limitations arising from differences in ^1^H-MRS methodology and sample demographics. We argue that more consistent methods and greater emphasis on phenotype studies will advance understanding of underlying cortical metabolite disturbance in ASD, and the detection, diagnosis, and treatment of ASD and other multi-dimensional psychiatric disorders.

## 1. Background

Autism spectrum disorder (ASD) encompasses a triad of abnormalities: social interaction, language and communication, and restricted and repetitive behaviors. The most recent revision of ASD in the Diagnostic and Statistical Manual (APA, 2013, DSM-5) includes all pervasive developmental disorders and Asperger's syndrome (AS). While the DSM-5 highlights the spectrum nature of ASD by removing the specification of language delay or disorder, by adopting this uni-dimensional diagnostic style it omits the inherent, multi-dimensional nature of ASD (APA, 2013). In fact, the ASD triad has been reported as genetically heterogeneous (Happé et al., 2006; Ronald et al., 2006; Robinson et al., 2012) Furthermore, the spectrum of the symptom triad, particularly social cognitive domain, are identified in the general population (Baron-Cohen et al., 2001; Ruzich et al., 2015). Ambiguity in diagnosis and treatment (Coolidge et al., 2013; Ford and Crewther, 2014), and group classification in scientific research has arisen as a result.

Current research methods fail to take into account the full extent of the spectrum heterogeneity of ASD, thus lack the specificity required to make conclusive inferences. This is illustrated in neuroimaging studies that use techniques such as magnetic resonance spectroscopy (MRS) which identifies abnormalities in molecular behavior related to ASD. We suggest that greater emphasis on research into ASD's specific phenotypes may resolve some of these limitations, helping to inform the theoretical framework around the ASD literature.

**Figure 1:**
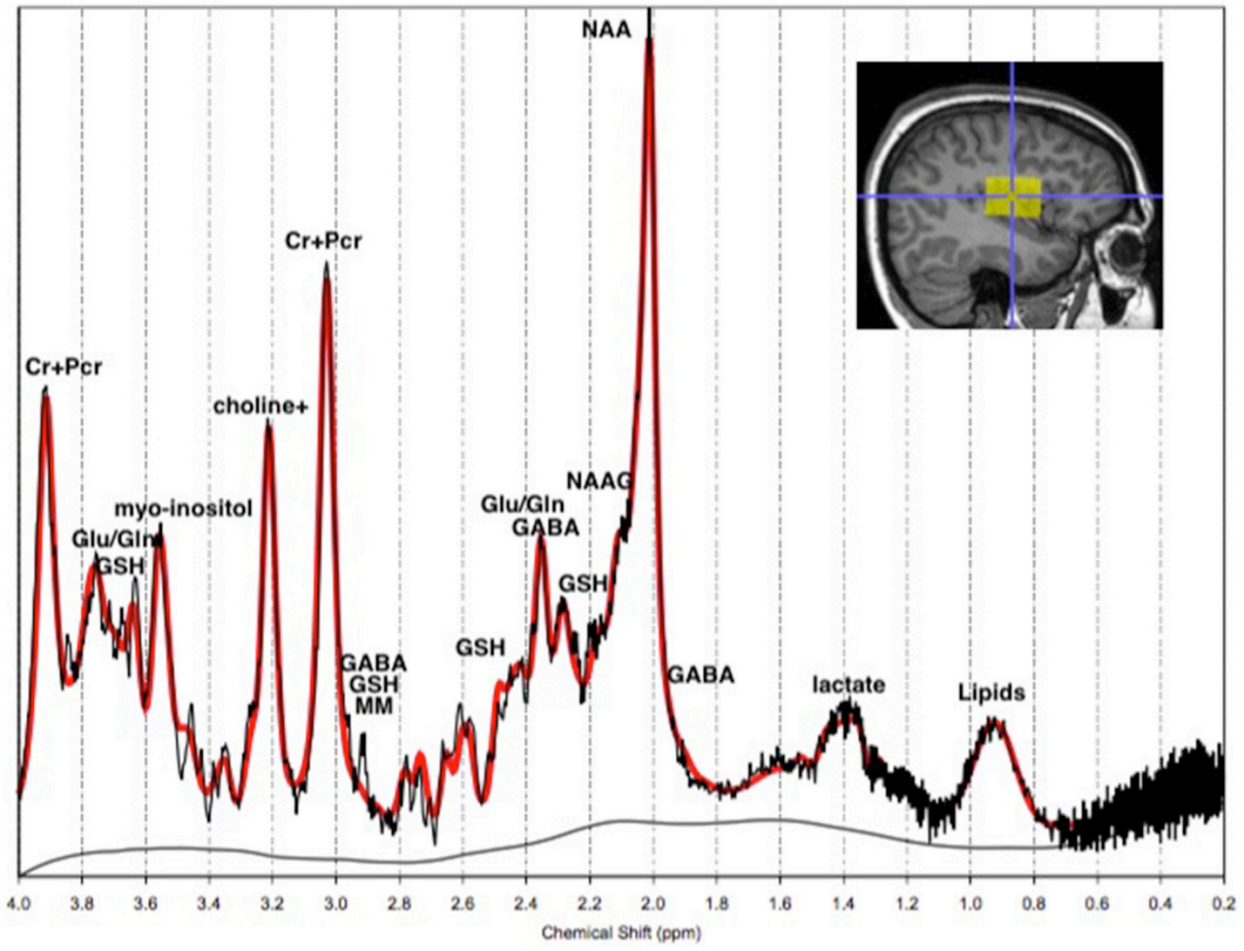
**The ^1^H-MRS chemical shift *in vivo***. The ^1^H-MRS chemical shift of a normal adult left temporal lobe at 3T. The y-axis represents the detected concentration or intensity of the metabolite in moles per liter of tissue, or millimolar (mM). The x-axis is the frequency chemical shift in parts per million (ppm), upon which metabolites are specified. Note: choline+, total Choline; Cr+PCr, creatine+phosphocreatine; Gln, glutamine; Glu, glutamate; GSH, glutathione; MM, macromolecules; NAA, N-acetyl-aspartyl; NAAG, NAA-glutamic acid.

MRS uses similar principles as magnetic resonance imaging (MRI), in that it is governed by the Larmor equation, ω = −γB_0_ where B_0_ is the external magnetic field and γ is a constant of a specific nucleus, known as the gyromagnetic ratio (Bertholdo et al., 2013; Kousi et al., 2013; Juchem and Rothman, 2014). During exposure to a magnetic field, the resonance of the atomic spins within nuclei become polarized in response to the field. In the event of a radio frequency (RF) pulse in MRS, spins within molecules absorb the energy and polarize with the RF field. Following the termination of the RF pulse, the spins precess along the axis of the magnet, creating a rotating magnetic field at the Larmor frequency. This induces an oscillating voltage in the RF receiver coil, which is being analyzed by the MR spectrometer (Juchem and Rothman, 2014). Each molecule has a different frequency shift due to its unique molecular environment. The frequency shifts are based on the chemical environment around the atomic nucleus, resulting in the “chemical shift” of a particular metabolite that is presented on a spectrum as shown in Figure 1 (Agzarian and Walls, 2011; Bertholdo et al., 2013; Kousi et al., 2013; Juchem and Rothman, 2014). Put simply, the chemical shift is the change of the atomic nucleus' MR frequency due to the shielding provided by the surrounding electrons. Due to the role of the external magnetic field, i.e. MR scanner strength, on the resonant frequency of the atomic nuclei, the chemical shift is expressed as a ratio-metric difference relative to a reference frequency ω_*ref*_. The chemical shift ($\delta =\frac{\omega -\omega_{ref}}{\omega_{ref}}$) is therefore independent of the applied external magnetic field, and is reported in parts per million (ppm; Ross and Bluml, 2001; Bertholdo et al., 2013; Juchem and Rothman, 2014).

**Figure 2:**
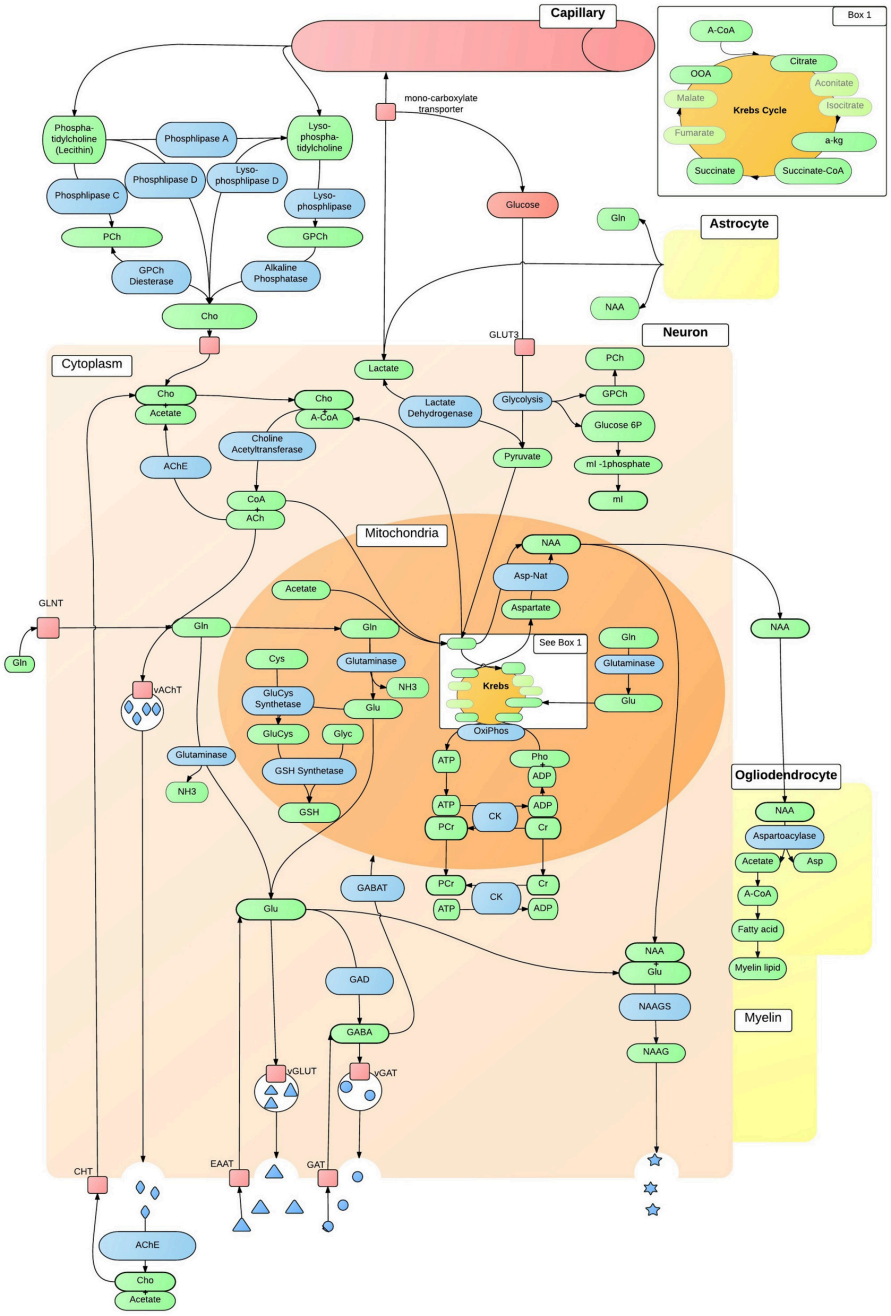
***In vivo* neuron metabolite interactions**. The intricate and complex interaction of metabolic pathways within a generic neuron *in vivo*, that are accessible to ^1^H-MRS. ACh, Acetylcholine; AChE, ACh Esterase; A-CoA, Acetyl-CoA; ADP, Adenosine Diphosphate; Asp, Aspartate; Asp-Nat, L-aspartate N-Acetyltransferase; ATP, Adenosine Triphosphate; Cho, Choline; ChT, Choline Transporter; CK, Creatine Kinase; CoA, Coenzyme A; Cr, Creatine; Cys, Cystine; EAAT, Excitatory Amino-Acid Transporter; GABA, γ-AminoButyric Acid; GABAT, GABA Transaminase; GAD, Glutamate Decarboxylase; GAT, Glutamate Transporter; Gln, Glutamine; GLNT, Glutamine Transporter; Glu, Glutamate; GluCys, γ-GlutamylCysteine Synthetase; GLUT3, Glucose Transporter 3; Glyc, Glycine; GPCh, Glycerophosphocholine Diesterase; GSH, Glutathione; mI, myo-Inositol; NAA, N-Acetylaspartate; NAAG, NAA-Glutamic Acid; NAAGS, NAAGS acid Synthase; NH_3_, Ammonia; OxiPhos, Oxidative Phosphorylation; PCh, Phosphocholine; PCr, Phosphocreatine; Pho, Phosphate; vAChT, Vesicular ACh Transporter; vGAT, Vesicular GABA Transporter; vGLUT, Vesicular Glutamate Transporter.

**Figure 3:**
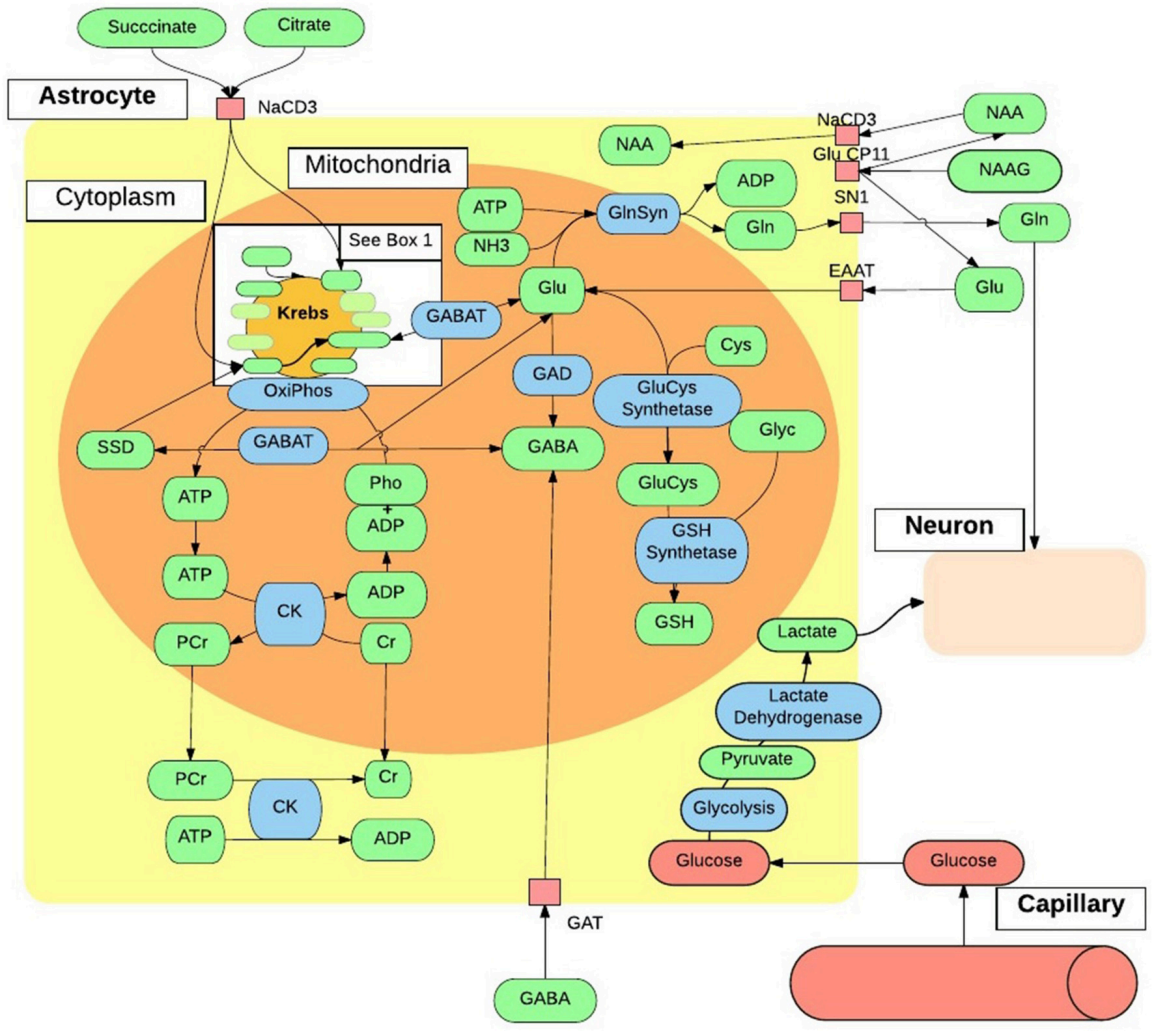
***In vivo* astrocytes metabolite interactions**. The intricate and complex interaction of metabolic pathways within a generic astrocyte *in vivo*, that are accessible to ^1^H-MRS. ADP, Adenosine Diphosphate; ATP, Adenosine Triphosphate; Cho, Choline; CK, Creatine Kinase; Cr, Creatine; Cys, Cystine; EAAT, Excitatory Amino-Acid Transporter; GABA, γ-AminoButyric Acid; GABAT, GABA Transaminase; GAT, Glutamate Transporter; Gln, Glutamine; GlnSyn, Glutamine Synthetase; Glu, Glutamate; GluCys, γ-GlutamylCysteine Synthetase; Glyc, Glycine; GSH, Glutathione; mI, myo-Inositol; NAA, N-Acetyaspartate; NAAG, NAA-Glutamic Acid; NH_3_, Ammonia; OxiPhos, Oxidative Phosphorylation; PCr, Phosphocreatine; Pho, Phosphate; SSD, Succinate-Semialdehyde Dehydrogenase.

Neurochemicals are molecules that are involved in cortical activity, and neurochemicals that are involved in, or are a product of, metabolic processes are metabolites. Each metabolite has a unique chemical shift which acts as its signature. This signature is used for the quantification of that metabolite. The most common method of metabolite quantification *in vivo* is through the proton resonance of hydrogen (^1^H) atoms (Bertholdo et al., 2013; Juchem and Rothman, 2014). ^1^H-MRS identifies many metabolites *in vivo*, although only reliably quantifies the low-molecular-weight metabolites: creatine and phosphocreatine (Cr+PCr), N-acetylaspartate (NAA), choline, myo-Inositol and lactate (Govindaraju et al., 1998). For this reason and at this stage of the technological advancement, the literature is limited to the aforementioned metabolites, and will be the focus of this review. Metabolites have intricate and complex interactions with other metabolites, as well as enzymes and neurotransmitters as illustrated in Figures 2, 3, which then translate to the interactions between aspects of human behavior and functioning. The quantification of a particular metabolite therefore depends on a number of processes, so reference concentrations of NAA, Cr+PCr or water allow for the calculation of each metabolites contribution to the spectra (Juchem and Rothman, 2014).

A given metabolite is represented by one or several signals along the MR chemical shift spectrum depending on the number of different proton environments within its molecular structure. These signals may be represented by a single peak, or a doublet, triplet or multiplet as a result of spin-spin coupling (J-coupling). J-coupling occurs when a proton within a molecule has neighboring protons, and the number of peaks is a function of the number of neighboring protons within the molecular structure (Govindaraju et al., 1998, 2000; Bertholdo et al., 2013; Juchem and Rothman, 2014). The area under the peak represents the quantity of the respective metabolite (see Figure 1). Furthermore, depending on the chemical composition of some metabolites, there may be overlap between them, making these metabolites difficult to isolate. This is true of GABA, glutamate and glutamine which are often quantified together as Glx (Govindaraju et al., 2000; Bertholdo et al., 2013; Kousi et al., 2013). This high level of complexity and interaction has broader implications for the theory of psychopathology. With advancing methods, ^1^H-MRS is an important tool for providing insight into the integrity of neuronal and glial cells, and cerebral energy metabolism in healthy and diseased brains. Nevertheless, researchers must be mindful of its limitations, such as the inability to isolate intracellular tissues (neurons, glia) from extracellular fluid metabolite levels, when making inferences based on the literature at large, due to inconsistencies highlighted in this review.

In psychopathological research, ^1^H-MRS elucidates possible neurochemical underpinnings of symptom phenotypes. Such markers have been investigated in ASD, with metabolite levels shown to differ between clinical and control samples (Murphy et al., 2002; Sokol et al., 2002; Kleinhans et al., 2007; Oner et al., 2007; Suzuki et al., 2010; Brown et al., 2013; Horder et al., 2013; Doyle-Thomas et al., 2014; Tebartz van Elst et al., 2014). Metabolite levels have also been shown to correlate with specific ASD phenotypes, for example; glutamate levels related to sensory sensitivity and social communication and interaction (Hardan et al., 2008; Doyle-Thomas et al., 2014; Tebartz van Elst et al., 2014), and NAA levels related to deficits in communication and social responsiveness (Kleinhans et al., 2009; Brown et al., 2013). However, behavioral data is often limited to the ASD sample despite presence of ASD traits amongst control populations (Baron-Cohen et al., 2001). The role of metabolites in ASD trait phenotypes is therefore largely unknown, and limits the interpretation of the literature at large. Differences in cognitive ability are also often overlooked in controls despite its association with NAA and choline levels (Jung et al., 1999). Demographic and methodological variation in ASD ^1^H-MRS studies are highlighted in Tables 1, 2, respectively.

**Table 1:** **Demographics for child and adult ASD ^1^H-MRS studies**.

**CHILDREN**		**N ASD (m:f)**	**N Cont (m:f)**	**Age ASD (range or SD)**	**Age Cont (range or SD)**	**IQ ASD (range or SD)**	**IQ Cont (range or SD)**	**Cont history**	**Cont behav**	**ASD Comorbid**	**ASD Medication**	**Sedation**
Bejjani et al., 2012	Exp1	8 (7:1)	10 (5:5)	11.2 (7.8–15.9)	13.2 (7.4–16.5)	90 (74–105)	112.3 (83–137)	Yes	NR		1xSSRI; 2xst	ASD 1xpr
	Exp2	26 (19:7)	16 (11:5)	10.2 (6.1–17.5)	11.8 (7.3–16.6)	106.6 (76–142)	101.2 (665–118)	Yes	NR		5xst; 1xnri; 1xac; 2xad; 1xap	No
Chugani et al., 1999		9 (8:1)	5 (4:1)	5.7 (3–12)	9 (6–14)	NR	NR	NR	NR	PNR	NR	NR
Corrigan et al., 2013	3–4 y	45 (38:7)	10 (8:2)	4 (3–4)	3.8 (3–4)	NR	NR	NR	Yes	No	1xst	ASD 45xpr
	6–7 y	31 (24:7)	18 (10:8)	6.6 (6–7)	6.6 (6–7)	NR	NR	NR	Yes	No	1xst; 5xad; 1xac	ASD 30xpr
	9–10 y	29 (24:5)	29 (25:4)	9.6 (9–10)	9.6 (9–10)	NR	NR	NR	Yes	No	6xst; 9xad; 6xap; 2xac	ASD 27xpr
Endo et al., 2007	ASD	12 (10:2)	16 (10:6)	13.1	11.5 (6–19)	84 (17.1)	95.1 (9.4)	Yes	No	No	1xap; 2xSSRI	NR
	AS	15 (12:3)		13.3		97 (15.4)		Yes	No	No	2 week	NR
	PDD	11 (10:1)		12		86 (15.9)		Yes	No	No	washout	NR
Fayed and Modrego, 2005		21 (18:3)	12 (5:7)	7.3 (4.1)	7.7 (0.3)	5 < 80	NR					
Friedman et al., 2003		45 (38:7)	13 (12:1)	47.4 m (3–4)	44.54 m(3–4)	NR	NR	Yes	Yes	PNR	No	ASD 45xpr; Cont 8xdi
Friedman et al., 2006	ASD	29 (26:3)	10 (8:2)	46.6 m	46.6 m (3–4)	NR	NR	Yes	Yes	PNR	No	ASD 29xpr; Cont 8xbe
	PDD	16 (12:4)		48.2 m								
	Total	45 (38:7)		47.4 m (3–4)								
Fujii et al., 2010	ACC	31 (25:6)	28 (21:5)	6.1 (2–13)	6.8 (2–15)	20–85+	NR	NR	No	NR	NR	NR
	DLPFC	20 (17:3)	18 (16:2)	7.5 (2–13)	7.8 (2–15)	20–85+	NR	NR	No	NR	NR	NR
Gabis et al., 2008		13 (10:3)	8 (3:5)	10 (7–16)	11 (7–17)	101 (15.6)	128 (16.1)	Yes	No	No	No	No
Hardan et al., 2008		18 (18:0)	16	(8–15)	(8–15)	93.8 (17.6)	116.8 (12.7)	Yes	Yes	PNR	NR	NR
Hashimoto et al., 1997		28 (20:8)	25 (16:9)	5.7 (2.8–12.2)	6.5 (2–13.8)	59.8 (21–88)	NR	NR	Yes	21.4% epilepsy	NR	<6 y tr
Hashimoto et al., 1998		23 (19:4)	34 (22:12)	7.9 (4.7–19.1)	11.1 (4.5–20)	NR	NR	NR	Yes	21.7% epilepsy	NR	<6 y tr
Hassan et al., 2013		10 (6:4)	10 (5:5)	11.4 (6–14)	11.3 (6–14)	NR	NR	Yes	NR	No	NR	NR
Hisaoka et al., 2001		55 (47:8)	51 (26:25)	5.8 (2–21)	6.2 (3 m–15)	NR	NR	NR	NR	NR	NR	NR
Kubas et al., 2012		12 (7:5)	16 (9:7)	10.55 (8–15)	11.35 (7–17)	NR	NR	NR	No	NR	NR	NR
Levitt et al., 2003		22 (18:4)	20 (10:10)	10.4 (5–16)	11.7 (5–16)	95 (18.3)	118.4 (16.2)	No	NR	PNR	5xSSRI; 1xap; 5xst; 2xsym; 1xac	ASD 10xpr
O'Brien et al., 2010	Total	22 (18:4)	22 (20:2)	23 (10–46)	23 (10–50)	101 (22)	110 (12)	Yes	No		No	
	Children	12 (11:1)	10 (9:1)	13 (10–16)	13 (10–16)	98 (24)	109 (6)					
Otsuka et al., 1999		27 (21:6)	10 (4:6)	2–18	6–14	NR	NR	NR	No	PNR	NR	NR
Sokol et al., 2002		10 (9:1)	NR	5.3 (2–12)	NR	NR	NR	NR	NR	PNR	3xac; 2xap; 1xst; 2xsym; 1xach	NR
Vasconcelos et al., 2008		10 (10:0)	10 (10:0)	9.5 (1.8)	8.5 (1.42)	NR	NR	Yes	No	No	2xSSRI; 2xap	ASD 9xse
Zeegers et al., 2007	ASD	17 (17:0)	NR	43 m (18 m–7 y)	NR	DQ 44 (8)	NR	NR	NR		1xac; 2xap	ASD se
MR & LD	PDD	8 (8:0)		45 m (18 m–7 y)	NR	DQ 80 (13)	NR	NR	NR			ASD se
DeVito et al., 2007		26 (26:0)	29 (29:0)	9.8 (6–17)	11.1 (6–16)	102.3 (14.8)	106 (11.4)	Yes	No	PNR	9xst; 5xap; 4xSSRI; 1xac	ASD 18xmi
Doyle-Thomas et al., 2014		20 (15:5)	16 (8:8)	11.5 (7–18)	12.5 (7–18)	99 (70–130)	100 (78–128)	Yes	No	No	NR	NR
Gaetz et al., 2014		17 (16:1)	17 (11:6)	11.5 (2.7)	13.3 (2.87)	88% >av; 35% MLI	NR	Yes (PNR)	No	PNR	2xSSRI; 1xmd; 1xap	NR
Harada et al., 2011		12	10	5.2 (2–11)	5.9 (3–12)	NR	NR	NR	NR	PNR	NR	ASD 10xtr; Cont 9xtr
Rojas et al., 2011		17 (14:3)	17 (8:9)	14 (5.2)	12 (5.2)	105.1 (15.5)	113.1 (12.1)	Yes	Yes	No	4xSSRI; 1xap	No
Joshi et al., 2013		7 (7:0)	7 (7:0)	14 (12–17)	12–17	108 (85–127)	>85	NR	NR	3xAnx; 3xADHD+Anx; 1xmd	1xSSRI; 3xst, ad, ap	NR
**ADULTS**												
Horder et al., 2013		28 (26:2)	14 (11:3)	28	34 (8.8)	100	107 (21)	Yes	No	No	No	No
	Narrow	15 (14:1)		29 (6)		95 (13)						
	Broad	13 (12:1)		27 (6.4)		103 (16)						
Kleinhans et al., 2007		13 (13:0)	13 (13:0)	24.5 (15–44)	22.5 (16–43)	99 (83–117)	115 (88–130)	Yes	No	No	1xSSRI; 1xst	No
Kleinhans et al., 2009		20 (18:2)	19 (17:2)	23.6 (6.6)	23.3 (5.2)	113.3 (14.2)	112.1 (15.2)	NR	Yes	No	NR	No
Murphy et al., 2002		14 (14:0)	18 (18:0)	30 (9)	32 (5)	97 (14)	102 (8)	Yes	Yes	NR	No	No
O'Brien et al., 2010	Adults	10 (7:3)	12 (11:1)	35 (20–50)	32 (20–50)	105 (15)	112 (15)	Yes	No	No	No	No
Oner et al., 2007		14 (14:0)	21 (21:0)	24.3 (17–38)	25 (17–38)	87.2 (10.9)	90.1 (11.7)	Yes	No	No	6xap	No
Page et al., 2006	AS	25 (20:5)	21 (16:5)	35.6 (11.5)	34.4 (9.3)	101 (16)	110 (17)	Yes	No	No	No	No
Suzuki et al., 2010		12 (12:0)	12 (12:0)	22 (18–25)	22.3 (19–24)	96.3 (71–117)	105.2 (85–125)	Yes	No	No	No	No
Bernardi et al., 2011		14 (12:2)	14 (12:3)	29 (21–50)	29 (21–50)	115 (14)	111 (16)	Yes	No	No	No	No
Brown et al., 2013		13 (9:4)	15 (6:9)	36.9 (25–48)	41.1 (25–48)	103.6 (16.3)	114.7 (11.8)	Yes	Yes	No	5xSSRI; 2xap; 1xnri	No
Tebartz van Elst et al., 2014		29 (19:10)	29 (19:10)	35.3 (9.1)	35.8 (8.5)	125.1 (12)	125 (13.4)	Yes	Yes	No	12xSSRI; 4xap	No

*1.5T, 3T, 4T; Anx, anxiety; AS, Asperger's Syndrome; PDD, Pervasive Developmental Disorder; Cont, Control; IQ, Intelligent Quotient; DQ, Development Quotient; MLI, Mild Language Impairment; ac, anti-convulsant; ach, anticholinergic; ad, anti-depressants; ap, anti-psychotic; nri, norepinephrine reuptake inhibitor; st, stimulants; SSRI, serotonin reuptake inhibitor; sym, sympatholytic; md, mood disorder; pr, propofol; be, benadryl; tr, tricloryl; di, diphenhydramine; mi, midazolam; se, sevofluran; NR, Not Reported; PNR, Psychiatric Data Not Reported*.

**Table 2:** **Scanning methods for child and adult ASD ^1^H-MRS studies**.

**CHILDREN**	**Scanner strength**	**Pulse sequence**	**Water suppression**	**Tissue comp**	**Diff**	**NAA**	**Cr+PCr**	**Cho**	**mI**	**Glx**	**Glu**	**GABA**	**Lac**
Bejjani et al., 2012	1.5T	PRESS (25/1500)	Yes			+	+	+	+	+			
			No	Yes	GM ACC	+	+	+	+	+			
Chugani et al., 1999	1.5T	PEPSI (30/2000)	No	No		+							+
Corrigan et al., 2013	1.5T	PEPSI (20/2000)	Yes (272/20/2000)	No		+	+	+	+	+			
Endo et al., 2007	1.5T	PRESS (35/2000)	CHESS	Yes	NS	+		+					
Fayed and Modrego, 2005	1.5T	PRESS (30/2000)		Yes	NS	+		+	+				
Friedman et al., 2003	1.5T	PEPSI (20/2000 272/2000)	No	No	Corr	+	+	+	+				
Friedman et al., 2006	1.5T	PEPSI (20/2000 272/2000)	No	No	Corr	+	+	+	+				
Fujii et al., 2010	1.5T	PRESS (135/1300)	No	No		+	+	+	+				
Gabis et al., 2008	1.5T	PRESS (40/2000)	No	No		+		+	+				
Hardan et al., 2008	1.5T	STEAM (20/1600)	No	Yes	NS	+	+		+		+		
Hashimoto et al., 1997	1.5T	STEAM (270/1500)	CHESS	No		+	+	+					+
Hashimoto et al., 1998	1.5T	STEAM (270/1500)	CHESS	No		+	+	+					+
Hassan et al., 2013	1.5T	PRESS (30/1500)	Yes	No							+		
Hisaoka et al., 2001	1.5T	PRESS (135/1300)	No	No		+	+	+					
Kubas et al., 2012	1.5T	PRESS (35/1500)	MOIST	No		+	+	+	+	+		+	
Levitt et al., 2003	1.5T	3D Axial (272/2300)	Yes	Yes	WM occ GM oar	+	+	+					
O'Brien et al., 2010	1.5T	PRESS (35/3000)	Yes	Yes	NS	+	+	+	+				
Otsuka et al., 1999	1.5T	STEAM (18/5000)	CHESS	No		+	+	+					
Sokol et al., 2002	1.5T	PRESS (NR)	NR	Yes		+	+	+	+				
Vasconcelos et al., 2008	1.5T	PRESS (30/1500)	NR	No		+	+	+	+				
Zeegers et al., 2007	1.5T	PRESS (144/2000)	Yes			+	+	+					
DeVito et al., 2007	3T	SEMS (135/1800)	CHESS	Yes	NS	+	+	+	+	+			
Doyle-Thomas et al., 2014	3T	2D axial (30/2000)	No	No		+	+	+	+	+			
Gaetz et al., 2014	3T	MEGA-PRESS (68/1500)	No	Yes	NS							+	
Harada et al., 2011	3T	STEAM (15/5000) MEGA-PRESS (68/2500)	Yes	Yes	NS	+					+	+	
Rojas et al., 2011	3T	MEGA-PRESS (70/2500)	Yes	Yes	NS		+						
Joshi et al., 2013	4T	2D-JPRESS (30-250/2000)	No	No	Corr						+		
**ADULTS**													
Horder et al., 2013	1.5T	PRESS (30/3000)	No	Yes	NS	+	+	+	+	+			
Kleinhans et al., 2007	1.5T	PRESS (35/3000)	No	No		+							
Kleinhans et al., 2009	1.5T	PRESS (30/2000)	No	Yes	NS								
Murphy et al., 2002	1.5T	PRESS (136/2000)	CHESS	Yes	NS	+	+	+					
O'Brien et al., 2010	1.5T	PRESS (35/3000)	Yes	Yes	NS	+	+	+	+				
Oner et al., 2007	1.5T	PRESS (270/1500)	No	No		+	+	+					
Page et al., 2006	1.5T	PRESS (35/3000)	CHESS	Yes	NS	+	+	+	+	+			
Suzuki et al., 2010	1.5T	PRESS (144/1500)	CHESS	Yes	NS	+	+	+					
Bernardi et al., 2011	3T	PRESS (30/2000)	Yes	No		+	+	+	+	+			
Brown et al., 2013	3T	PRESS (30/2000)	No	No		+	+	+	+	+	+		
Tebartz van Elst et al., 2014	3T	PRESS (30/3000)	No	Yes	Corr	+	+	+	+	+	+		

*1.5T, 3T, 4T; Cho, Choline; Cr+PCr, Creatine+Phosphocreatine; GABA, γ-Aminobutyric Acid; Glx, Glutamine+Glutamate; Glu, Glutamate; Lac, Lactate; mI, myo-Inositol; NAA, N-Acetyl-Aspartate; CHESS, Chemical Shift Selective; PEPSI, Proton Echo Planar Spectroscopic Imaging; PRESS, Point-Resolved Spectroscopy; STEAM, Stimulated Echo Acquisition Mode; T, Testla; Corr, Corrected*.

Literature reviews to date highlight the importance of advancing ^1^H-MRS techniques in ASD research, showing generally decreased NAA (Aoki et al., 2012; Baruth et al., 2013), Cr+PCr, choline, mI and Glx (Baruth et al., 2013), and increased Glx in adults (Naaijen et al., 2015). However, there is a great deal of inconsistency in region of interest such that inferring about the efficacy of methodological advances is difficult (Baruth et al., 2013; Naaijen et al., 2015). Previous reviews also do not discuss the complexity and interactions within and between the metabolic pathways, which is of particular importance in development of the theoretical framework around ASD. Furthermore, recent reviews do not discuss the implications of omitting trait phenotype data across experimental groups, despite existing neurological and psychophysiological differences at a personality trait level (Gomot et al., 2008; Sutherland and Crewther, 2010; Dinsdale et al., 2013; Ford and Crewther, 2014). The current review focuses on these shortcomings and methodological inconsistencies across studies that inevitably compromises the understanding of metabolite abnormalities in ASD.

## 2. Creatine

Creatine and phosphocreatine levels are a reflection of cellular adenosine triphosphate (ATP) metabolism (Pouwels and Frahm, 1998; Rae, 2014). Creatine synthesis begins in the kidney where arginine and glycine produce guanidinacetate (GA) via arginine-glycine transaminase. GA is transported to the liver and creatine is synthesized via GA methyltransferase. Creatine is then transported to the brain as an essential component of energy equilibrium *in vivo* as illustrated in Figures 2, 3 (Ross and Bluml, 2001; Rae, 2014). Creatine and phosphocreatine play an essential role in ATP and adenosine diphosphate (ADP) energy transfer and equilibrium within cells. ATP results from oxidative phosphorylation in neuronal and glial mitochondria, and glycolysis in the cytosol. In order to store energy effectively, a phosphate bond is released from ATP and catabolised to ADP, via the enzyme creatine kinase. The free phosphate bond then binds with creatine to form phosphocreatine (Ross and Bluml, 2001; Kousi et al., 2013; Rae, 2014). When cellular mitochondria require energy, ADP and a third phosphate bond are resynthesized to ATP via oxidative phosphorylation. In the cytosol, the reversal of creatine kinase generates ATP; phosphocreatine releases a phosphate bond, resulting in creatine, which is taken up by ADP to resynthesize ATP. Due to the high expression of creatine in the mitochondria of neurons and in the cytosol of astrocytes (in Rae, 2014), creatine is most abundant in the cerebellum, followed by gray matter and white matter (Pouwels and Frahm, 1998; Ross and Bluml, 2001; Rae, 2014; Turner and Gant, 2014). Creatine and creatine kinase are essential in cellular energy metabolism and in the maintenance of cortical homeostasis, and may play a mobilizing role for myo-inositol (Ross and Bluml, 2001; Rae, 2014; Turner and Gant, 2014). Figures 2, 3 illustrate the importance of the cellular energy metabolism in the normal production and functioning of NAA, glutamate and GABA. Creatine concentration *in vivo* corresponds to local expression and activity of creatine kinase (Pouwels and Frahm, 1998; Rae, 2014).

In ^1^H-MRS, creatine and phosphocreatine (Cr+PCr) are quantified together at 3.03 and 3.93 ppm (Kousi et al., 2013), at a concentration of 5.1–10.6 mmol/kg_ww_ (Govindaraju et al., 2000). There is substantial variability of Cr+PCr across studies as demonstrated by the differences between ASD and control groups reported in Table 3, with Cr+PCr reduction reported across the cortex in children with ASD (Friedman et al., 2003; Levitt et al., 2003; DeVito et al., 2007; Hardan et al., 2008; Corrigan et al., 2013). By contrast, regional increases in Cr+PCr are reported in adults with ASD (Murphy et al., 2002; Page et al., 2006; Suzuki et al., 2010; Brown et al., 2013). Variable Cr+PCr levels are thought to indicate differences in energy systems and metabolism at an intracellular level (Bertholdo et al., 2013). Turner and Gant (2014) provide an extensive review of the biochemistry of creatine, reporting an association between reduced Cr+PCr and abnormal speech and motor learning, intellectual disability and ASD-like behaviors. It is suggested that these are symptoms of delayed or impaired axon growth during development, of which Cr+PCr is an important ingredient (Turner and Gant, 2014). Finding of this review largely support delayed or impaired axon growth across the cortex for children with ASD, as well as abnormalities in the storage and transport of cellular energy, and in regulating tissue energy (Turner and Gant, 2014). In adults with ASD however, Cr+PCr appears to accelerate to abnormally high levels within the auditory cortex, hippocampus-amygdala complex (HAC) and medial prefrontal region, suggesting greater availability and transportation of Cr+PCr (Murphy et al., 2002; Page et al., 2006; Suzuki et al., 2010; Brown et al., 2013).

**Table 3:** **^1^H-MRS quantified Creatine+Phosphocreatine (Cr+PCr) differences for children and Adults with ASD**.

**Region**				**Children**		**Adult**	**Region**				**Children**		**Adult**
GM			⇊	Corrigan et al., 2013^†^			HAC						Kleinhans et al., 2009
				Corrigan et al., 2013^‡^					Left		Zeegers et al., 2007	⇈	Suzuki et al., 2010
				Corrigan et al., 2013^#^					Right		O'Brien et al., 2010		O'Brien et al., 2010
				Friedman et al., 2006							Otsuka et al., 1999	⇈	Page et al., 2006
	Frontal			DeVito et al., 2007			Thalamus				Levitt et al., 2003		Bernardi et al., 2011
	Occipital	Left	⇊	DeVito et al., 2007					Left	⇊	Hardan et al., 2008		
		Right		DeVito et al., 2007						⇊	Friedman et al., 2003		
	Temporal	Left	⇊	DeVito et al., 2007					Right		Hardan et al., 2008		
		Right		DeVito et al., 2007							Friedman et al., 2003		
WM			⇊	Corrigan et al., 2013^†^									
			⇈	Corrigan et al., 2013^‡^			Cerebellum				DeVito et al., 2007		
									Left		Vasconcelos et al., 2008		Tebartz van Elst et al., 2014
				Corrigan et al., 2013^#^							Otsuka et al., 1999		
				Friedman et al., 2006					Right				Suzuki et al., 2010
				DeVito et al., 2007			Cingulate				Hisaoka et al., 2001		
	Frontal			Levitt et al., 2003							Friedman et al., 2003		
		Left	⇊	Friedman et al., 2003			Putamen				Levitt et al., 2003		
				Zeegers et al., 2007							Friedman et al., 2003		
		Right		Friedman et al., 2003			ACC				Levitt et al., 2003		Bernardi et al., 2011
	Parietal			Levitt et al., 2003							Vasconcelos et al., 2008		Tebartz van Elst et al., 2014
		Left	⇊	Friedman et al., 2003							Bejjani et al., 2012		
		Right		Friedman et al., 2003							Fujii et al., 2010		
Frontal				Hisaoka et al., 2001			Caudate				Friedman et al., 2003		
				Levitt et al., 2003				Head	Left		Levitt et al., 2003		
		Left		Vasconcelos et al., 2008					Right	⇈	Levitt et al., 2003		
	MPF	Right			⇈	Murphy et al., 2002		Body	Left	⇊	Levitt et al., 2003		
	DLPFC	Left		Fujii et al., 2010	⇊	Horder et al., 2013			Right		Levitt et al., 2003		
		Right		Fujii et al., 2010			Callosum	Ant		⇊	Friedman et al., 2003		
Parietal				Hisaoka et al., 2001				Post			Friedman et al., 2003		
				Levitt et al., 2003			Insula		Left	⇊	Friedman et al., 2003		
		Left				Horder et al., 2013			Right		Friedman et al., 2003		
		Right		Hashimoto et al., 1998		Page et al., 2006	Striatum		Left		Vasconcelos et al., 2008		
	MPF					Murphy et al., 2002	Brainstem				Hisaoka et al., 2001		
	IPS					Bernardi et al., 2011	BasalGanglia		Left			⇊	Horder et al., 2013
Temporal				Hisaoka et al., 2001									
	MTL			Friedman et al., 2003									
Auditory		Left		Rojas et al., 2011	⇈	Brown et al., 2013							
		Right			↑	Brown et al., 2013							
TPJ						Bernardi et al., 2011							
STG				Friedman et al., 2003									
Occipital				Friedman et al., 2003									
		Left		Levitt et al., 2003									
		Right	⇊	Levitt et al., 2003									

1.5T, 3T; ⇊, p < 0.001; ↓, p < 0.01; ⇈, p < 0.001; ↑, p < 0.01;

† 3–4 years;

‡ 6–7 years;

# 9–10 years;

*ACC, Anterior Cingulate Cortex; Ant, Anterior; Cent Semi, Centrum Semiovale; Cr+PCr, Creatine+Phosphocreatine; DLPFC, Dorsolateral Prefrontal Cortex; GM, Gray Matter; HAC, Hippocampus-Amygdala Complex; IPS, Intraparietal Suclus; MPF, Medial Prefrontal; MTL, Medial Temporal Lobe; Post, Posterior; STG, Superior Temporal Gyrus; TPJ, Temporo-parietal Junction; WM, White Matter*.

Despite group differences in Cr+PCr level, no relationship between Cr+PCr level and autism symptom severity has been found in children (Murphy et al., 2002; Page et al., 2006; Horder et al., 2013), nor with communication, social anxiety and social distress in adults (Kleinhans et al., 2009). Cr+PCr level in the HAC of adults with ASD decreased with more severe social deficits and repetitive behaviors (Kleinhans et al., 2009). Later still, hippocampal Cr+PCr in high functioning adults increased with more aggressive behavior (Suzuki et al., 2010). These correlates suggest that increased hippocampal cell density is implicated in aggressive behavior modulation (Suzuki et al., 2010).

Cortical Cr+PCr was in the past considered stable, and thus has been used extensively as a reference to other, more clinically variable metabolites (Ross and Bluml, 2001; Bertholdo et al., 2013; Brown et al., 2013; Kousi et al., 2013). However, this review demonstrates clinical differences in Cr+PCr levels (Murphy et al., 2002; Friedman et al., 2003; Levitt et al., 2003; Page et al., 2006; DeVito et al., 2007; Hardan et al., 2008; Suzuki et al., 2010; Brown et al., 2013; Corrigan et al., 2013), and recently the use of Cr+PCr has been criticized (Rae, 2014; Turner and Gant, 2014). Future research should avoid this method, and careful interpretation of existing literature employing a metabolite reference must ensue.

In sum, increased Cr+PCr is associated with greater cell density. Thus, perhaps counterintuitively, greater HAC and hippocampal cell density in adults with ASD may be related to more aggressive behavior, and less severe social deficits and repetitive behaviors, respectively. The role of creatine and phosphocreatine in ASD pathology, and clinical and nonclinical behavioral phenotypes, must be further scrutinized.

## 3. Glutamate, glutamine, γ-aminobutyric acid (GABA) and glutathione

Glutamate, glutamine, and GABA are amino acid neurotransmitters that interact through the glutamate/GABA-glutamine cycle to maintain cortical excitation/inhibition equilibrium (for a review see Rubenstein and Merzenich, 2003; Bak et al., 2006). Glutathione is synthesized from glutamate, cysteine, and glycine through γ-glutamylcysteine synthetase and GSH (glutathione transportation form) synthetase (Meister and Anderson, 1983). Their interconnectivity is illustrated in Figures 2, 3. Due to their structural and neurochemical similarity, glutamate, glutamine, GABA, and glutathione possess a similar resonant frequency, thus are difficult to isolate and quantify with standard ^1^H-MRS protocols at a low field strength of 1.5T (Puts and Edden, 2012; Rae, 2014), and are reported as Glx (DeVito et al., 2007; Bernardi et al., 2011; Bejjani et al., 2012; Corrigan et al., 2013; Horder et al., 2013; Kousi et al., 2013; Doyle-Thomas et al., 2014). However, these metabolites have vastly different functions and are implicated in different theories of psychopathology, including the hyper-glutamatergic (Fatemi, 2008), hypo-GABAergic (Fatemi et al., 2009) and N-methyl-D-aspartate receptor (NMDAr) dysfunction theories (Gandal et al., 2012; Lee et al., 2015). The methods by which these metabolites were quantified should therefore be taken into account when interpreting the broader literature.

An MR scanner strength of 3T or above and specific scanner parameter adjustments [echo time (TE): Schubert et al., 2004; Ganji et al., 2012, radio frequency pulse sequence: Hancu, 2009; Puts and Edden, 2012; Mullins et al., 2014] are recommended to isolate glutamate and glutamine, and are essential for the isolation of GABA and glutathione. *A priori* metabolite peak frequency information also improves its isolation (Govindaraju et al., 1998, 2000). Specialized software tools such as LCModel (Provencher, 2001), Tarquin (Wilson et al., 2011), and jMRUI (Naressi et al., 2001) are also utilized for metabolite quantification as they provide basis sets of predefined spectral peak models, rather than individual metabolite resonance values (Provencher, 2001; Mullins et al., 2014). Gannet (Edden et al., 2014) has been developed specifically for GABA-MRS analysis, with the frequency and phase correction specialized to deliver an accurate spectra while dealing with instability in the acquisition.

Aberrations in the glutamate-GABA-glutamine cycle in ASD will be discussed in turn below. Genetic influences on the neurobiological interaction between glutamate and GABA are beyond the scope of this review, see Pardo and Eberhart (2007) for a detailed review.

### 3.1. Glutamate-glutamine cycle

Glutamate is synthesized from glutamine in the mitochondria of glutamatergic neurons via the phosphate-activated enzyme glutaminase, which releases ammonia (Gladden, 2004; Bak et al., 2006; Amaral et al., 2013). Glutamate is taken up by a vesicular transporter and then released into the synapse. Any glutamate not taken up by the postsynaptic neuron is then returned to the presynaptic terminal or taken up by neighboring microglia and astrocytes to maintain a low concentration of glutamate in the synaptic cleft and avoid excitotoxicity (for an extensive review see McKenna, 2007). Within the astrocyte, glutamine synthetase catalyses glutamate and ammonia to resynthesize glutamine, as well as interacting with the Krebs cycle. Glutamine level is most abundant in the glia as a function of glutamate uptake (Bak et al., 2006) and is thus thought to predict glutamatergic activity (Rothman et al., 2003; Marsman et al., 2013). Glutamine is then transported back to the glutamatergic neuron, or may be resynthesized to glutamate via glutaminase in the astrocyte (Gladden, 2004; Bak et al., 2006; McKenna, 2007; Amaral et al., 2013).

Glutamatergic neurons are the most abundant cortical neurons, taking up 60–80% of glucose oxidation and energy consumption (Rothman et al., 2003). Glutamate is also the most abundant neurotransmitter (90% of synapses) (McKenna et al., 2011), playing an important role in neurodevelopmental processes such as neural migration, differentiation, plasticity (Page et al., 2006; Bejjani et al., 2012; Baruth et al., 2013), as well as metabolism through its contribution to the neuronal and glial Krebs cycle (Rae, 2014). Both glutamate and glutamine are most concentrated in gray matter (Pouwels and Frahm, 1998), and Glx interaction is involved in neurotransmitter regulation and detoxification (Kousi et al., 2013). Furthermore, NMDArs and enzyme synthesis modulate the availability of neuronal, glial and synaptic glutamate and glutamine, and go undetected in ^1^H-MRS, thus inherently affecting the level of detected Glx (Stagg et al., 2011).

In ^1^H-MRS, glutamate level is quantified from a prominent multiplet between 2.35 and 2.04 ppm, and a doublet-of-doublets at 3.74 ppm (Govindaraju et al., 2000). Glutamine level is quantified from a prominent multiplet between 2.46 and 2.12 ppm, and a triplet at 3.75 ppm (Govindaraju et al., 2000). The concentration of brain glutamate is between 6.0 and 12.5 mmol/kg_ww_, while glutamine concentration is between 3.0 and 5.8 mmol/kg_ww_ (Govindaraju et al., 2000). The interpretation of Glx level tends to focus on the excitatory nature of glutamate, overlooking the roles of glutamine (e.g. DeVito et al., 2007; Horder et al., 2013). Some research has shown no influence of glutamine on differences in Glx, suggesting that Glx may in fact be a relatively sound measure of glutamate (Stagg et al., 2009). However, due to the inter-relatedness of glutamate and glutamine, it is unclear what separate quantification of these metabolites might indicate.

Glutamate and Glx differences for children and adults with ASD are reported in Table 4. For children with ASD, at 4T, glutamate concentration is reduced in the auditory cortex and increased in the anterior cingulate cortex (ACC; Joshi et al., 2013). Increased glutamate is also reported in subcortical regions at 1.5T, which is more likely a measure of Glx (Hassan et al., 2013). Reduced Glx is reported throughout the brain at 3T (DeVito et al., 2007), which may be a result of reduced glutamate or glutamine, or both. No studies investigate isolated glutamine in children, although a blood plasma study reported increased glutamate and reduced glutamine in high functioning children with ASD (Shimmura et al., 2011). For adults with ASD, increased auditory cortical glutamate and Glx is reported (Brown et al., 2013), while in the ACC it is reduced (Tebartz van Elst et al., 2014). Additional differences in Glx are reported in Table 4.

**Table 4:** **^1^H-MRS quantified Glutamate (Glu) and Glx Differences for children and Adults with ASD**.

**Region**				**Glutamate**		**Children Glx**		**Glx/Cr+PCr**		**Glutamate**		**Adult Glx**		**Glx/Cr+PCr**
GM						Corrigan et al., 2013^†^^‡^^#^								
	Frontal				⇊	DeVito et al., 2007								
	Occipital				⇊	DeVito et al., 2007								
	Temporal				↓	DeVito et al., 2007								
WM						DeVito et al., 2007								
					⇊	Corrigan et al., 2013^†^								
						Corrigan et al., 2013^‡^^#^								
Frontal							⇊	Kubas et al., 2012						
		Left	⇈	Hassan et al., 2013										
				Harada et al., 2011										
	DLPFC	Left										Horder et al., 2013		
Parietal		Left										Horder et al., 2013		
		Right										Page et al., 2006		Page et al., 2006
IPS											↓	Bernardi et al., 2011		
Auditory									⇈	Brown et al., 2013	⇈	Brown et al., 2013		
		Left		Joshi et al., 2013										
		Right	↓	Joshi et al., 2013										
TPJ											↓	Bernardi et al., 2011		
HAC		Right									⇈	Page et al., 2006		Page et al., 2006
Thalamus						Hardan et al., 2008		Doyle-Thomas et al., 2014			↓	Bernardi et al., 2011		
Cerebellum					⇊	DeVito et al., 2007								
		Left	⇈	Hassan et al., 2013						Tebartz van Elst et al., 2014		Tebartz van Elst et al., 2014		
Putamen							⇈	Doyle-Thomas et al., 2014						
ACC			⇈	Joshi et al., 2013					⇊	Tebartz van Elst et al., 2014	⇊	Tebartz van Elst et al., 2014		
			⇈	Hassan et al., 2013										
		Left				Bejjani et al., 2012					↓	Bernardi et al., 2011		
		Right			⇈	Bejjani et al., 2012					⇊	Bernardi et al., 2011		
Caudate								Doyle-Thomas et al., 2014						
Lent		Left		Harada et al., 2011										
Striatum		Left	⇈	Hassan et al., 2013										
BasGan		Left									⇊	Horder et al., 2013		

1.5T, 3T, 4T; ⇊, p < 0.001; ↓, p < 0.01; ⇈, p < 0.001; ↑, p < 0.01;

† 3–4 years;

‡ 6–7 years;

# 9–10 years;

*ACC, Anterior Cingulate Cortex; BasGan, Basal Ganglia; Cr+PCr, Creatine+Phosphocreatine; DLPFC, Dorsolateral Prefrontal Cortex; Glx, Glutamate+Glutamine; GM, Gray Matter; HAC, Hippocampus-Amygdala Complex; IPS, Intraparietal Suclus; Lent, Lenticular Nuclei; TPJ, Temporo-parietal Junction; WM, White Matter*.

Across child and adult studies, higher and lower glutamate and Glx levels appear regionally specific. However, the ratio of glutamate to glutamine in the Glx level is unknown, thus so is the degree of excitatory neurotransmission. Furthermore, abnormalities in glutamate and Glx level in ASD appear to differ between adult and child samples, suggesting age or disease related changes throughout the lifespan (Naaijen et al., 2015). Tebartz van Elst et al. (2014) suggest an ACC over-excitation in children, and over-inhibition in adults with ASD. Their hypothesis was supported by finding similar glutamine levels between ASD and control adults (Tebartz van Elst et al., 2014), as well as no change in glutamine following excitatory and inhibitory stimulation (Stagg et al., 2009).

To date, no childhood studies report isolated glutamate correlates of ASD behavioral phenotypes. Reports of heightened sensory sensitivity and deficits in body movement modulation are associated with trend level thalamic Glx increase (Hardan et al., 2008). Increased thalamic Glx/Cr+PCr is also associated with poor social interaction in children with ASD (Doyle-Thomas et al., 2014). In adult studies, autism spectrum quotient (AQ) score increases with left auditory glutamate level (Brown et al., 2013), and with reduced ACC glutamate (Tebartz van Elst et al., 2014). Scores on AQ subscales Communication and Imagination, as well as empathy, also increases with decreasing glutamate level (Tebartz van Elst et al., 2014). This is also seen for Communication and Glx in the basal ganglia (Horder et al., 2013) and ACC (Tebartz van Elst et al., 2014). Alternately, control group ACC glutamate levels increase with social skill deficits and more fluid imagination (Tebartz van Elst et al., 2014), while Glx in the ACC reduces with more social skill deficits (Tebartz van Elst et al., 2014). Reduced dorsolateral prefrontal cortex (DLPFC) glutamate in controls is suggested to predict better perspective taking (Montag et al., 2008), and reduced Glx in the ACC may related to executive function deficits such as decision-making, impulse control, empathy and emotion (Bernardi et al., 2011). No relationship between ASD diagnostic domains and Glx in the HAC and parietal regions has been reported (Page et al., 2006).

In sum, regional differences in glutamate and Glx have been related to ASD related phenotypes and behavior, though much needs to be done to isolate glutamate and glutamine, as well as clinical and non-clinical phenotypes, in order to draw meaningful conclusions.

### 3.2. GABA-glutamate-glutamine cycle

GABA is the major inhibitory neurotransmitter in the cortex, responsible for halting excitatory glutamatergic activity, so naturally, disruption to either of these metabolites will affect the other (Marsman et al., 2013). GABAergic inter-neurons make up 15–20% of cortical neurons (Buzaki et al., 2007). GABA level is low at 1.3 to 1.9 mmol/kg_ww_ (Govindaraju et al., 2000), with ~1 mmol/kg_ww_ in intracellular space and ~2μ mol/kg_ww_ in extracellular space (Puts and Edden, 2012; Rae, 2014). In GABAergic inter-neurons, glutamine is synthesized to glutamate via glutamate synthase, and is then synthesized via the rate limiting enzyme glutamate decarboxylase (GAD) 67 into GABA before transportation and release at the synapse (Bak et al., 2006). GABA receptors on the postsynaptic neuron receive GABAergic neurotransmitters, while ~20% is taken up by neighboring astrocytes, suggesting a role in the modulation of GABAergic synapses (Rae, 2014). Any excess GABA returns to the extrasynaptic membrane of the presynaptic terminal, or is taken up by neighboring glia to control overspill (Rae, 2014). In the glia, GABA transaminase metabolizes GABA and α-ketoglutarate to form succinic semialdehyde. The succinic semialdehyde is oxidized to re-enter the Krebs cycle as succinate. Succinate is also taken up by available α-ketoglutarate to reform glutamate (Bak et al., 2006; Rae, 2014).

The inhibitory role of GABA is thought to be adult brain specific, with GABA and its transporters involved in the maintenance and modulation of cognition, sleep, motor control, pain and anxiety (Rae, 2014). The role of GABA in children, however, is initially excitatory in early developmental periods, and switches to inhibitory via the chloride potassium co-transporter (Herlenius and Lagercrantz, 2004; Ben-Ari et al., 2007; Quattrocki and Friston, 2014). Aberrations in this transition may result in behavioral abnormalities from early development, which is supported by studies that demonstrate the excitatory role of GABA in the neurodevelopmental stages preceding glutamate maturation (for extensive reviews, see Hensch, 2005; Ben-Ari et al., 2007; LeBlanc and Fagiolini, 2011).

GABA resonates at 1.9, 2.3, and 3.0 ppm, and requires editing pulse sequences such as MEGA-PRESS (Meshcher-Garwood point resolved spectroscopy) to quantify its peak at 1.9 ppm (Mullins et al., 2014). Stagg et al. (2011) suggests the quantification of GABA is in fact more reliable than that of glutamate, however this only applies when GABA is acquired with isolation techniques. Macromolecules (MMs) such as DNA, RNA, most proteins and phospholipids (Ross and Bluml, 2001) contribute GABA, glutamate and glutamine levels (Ganji et al., 2012; Mullins et al., 2014; Rae, 2014) and are inaccessible with NMR and thus difficult to exclude (Mullins et al., 2014). MMs however, should be addressed in reports (Bhattacharyya, 2014).

Investigations into GABA levels in ASD are limited due to the relatively recent development of specialized GABA ^1^H-MRS protocols, with only childhood studies published. Table 5 illustrates a general reaction in GABA level between children with ASD and controls (Kubas et al., 2012), however data were not acquires with a specialized ^1^H-MRS methods (see Table 1), and therefore may therefore reflect Glx decrease. Nonetheless, auditory cortex GABA was not related to language function or social responsiveness in children with ASD (Gaetz et al., 2014). In sum, due to the overall lack of research in this area, little is known about the implications of GABA aberrance in ASD, though differences suggest abnormalities in inhibitory control pathways. Further research with phenotype data is essential for the understanding of inhibitory modulation in the ASD triad.

**Table 5:** **^1^H-MRS quantified GABA differences for children with ASD**.

**Region**			**GABA**		**GABA/Ratio**	
Frontal				↓	/Cr+PCr	Kubas et al., 2012
	Left	↓	Harada et al., 2011	↓	/NAA	Harada et al., 2011
					/Glu	Harada et al., 2011
Motor	Left			↓	/Cr	Gaetz et al., 2014
Auditory	Left			↓	/Cr	Gaetz et al., 2014
				↓	/Cr	Rojas et al., 2011
Visual	Left				/Cr	Gaetz et al., 2014
Lent	Left				/NAA	Harada et al., 2011
					/Glu	Harada et al., 2011

*1.5T, 3T; ⇊, p < 0.001; ↓, p < 0.01; ⇈, p < 0.001; ↑, p < 0.01 Cr+PCr, Creatine+Phosphocreatine; GABA, γ-Aminobutyric Acid; Glu, Glutamate; Lent, Lenticular Nuclei; NAA, N-Acetyl-Aspartate*.

### 3.3. Excitatory/inhibitory equilibrium

Due to their interconnectivity (Figures 2, 3), reduced GABA along with regional differences in glutamate and Glx concentration might indicate a cortical excitation/inhibition imbalance, or a disruption in synaptic mechanisms, which then contributes to ASD psychopathology (Rojas et al., 2011; Parellada et al., 2014). Furthermore, the glutamate/GABA cycle is involved in the production of the neurotransmitter NAA-glutamic acid (NAAG) and in the Krebs cycle (Figures 2, 3). Regional increases of glutamate supports Fatemi's (2008) hyper-glutamatergic hypothesis that the GAD67 rate limiting enzyme is deficient and results in reduced synthesis of glutamate to GABA (Fatemi, 2008; Bejjani et al., 2012), thus a higher concentration of glutamate (Blaylock and Strunecka, 2009; Shimmura et al., 2011; Hassan et al., 2013). Stagg et al. (2009) suggest GAD67 dysfunction might be caused by a deficiency in glutamate modulation. Cortical GABA concentration is low however (Govindaraju et al., 2000), and may not have a substantial influence on glutamate levels. Increased glutamate has also been thought to result from reduced glutamine synthetase or increased glial population in ASD (Fatemi, 2008; McKenna et al., 2011). Although, post-synaptic receptors of GABA may up-regulate to compensate for reduced GABA inhibition (Benes et al., 1996), reduced GABA may be a result of GABA_A_ receptor down-regulation (Fatemi, 2008; Fatemi et al., 2009; Gaetz et al., 2014). This down regulation may lead to glutamatergic hyper-function (Harada et al., 2011) and disrupt the maintenance and modulation of excitatory/inhibitory equilibrium.

Theories regarding the role of NMDArs in glutamate, glutamine and GABA levels are inconclusive. The uptake of glutamate by NMDArs triggers the flux of calcium into the cell, facilitating synaptic plasticity that is necessary for memory and learning (Debanne et al., 2003; Lally et al., 2014). NMDAr antagonism leads to disinhibition of excitatory pyramidal cells (Lisman et al., 2008), as well as reduced excitation of GABAergic neurons (Kondziella et al., 2007; Marsman et al., 2014). Altogether, an disinhibited excitatory cells along with insufficient inhibitory output might facilitate excitotoxicity, which may then lead to cell death through the influx of calcium and downstream behavioral effects (Rossignol, 2011). Indeed, NMDAr down-regulation in has been associated with autism and schizophrenia-like behaviors and electrophysiology (Rossignol, 2011; Gandal et al., 2012).

Excitotoxicity is especially dangerous during the critical developmental stage in pre- and early postnatal development (Deng et al., 2004; LeBlanc and Fagiolini, 2011), with excitatory/inhibitory imbalance affecting cortical plasticity (Lam et al., 2006; LeBlanc and Fagiolini, 2011; Berger et al., 2013). Autistic symptoms arise in this critical period, providing strong evidence for an excitation/inhibition imbalance that leads to aberrant neuronal growth and connectivity (Harada et al., 2011; LeBlanc and Fagiolini, 2011). However, this association does not account for possible compensatory up-regulation of GABA receptors (Benes et al., 1996), and due to the excitatory role of GABA during this stage (Herlenius and Lagercrantz, 2004; Ben-Ari et al., 2007; Rossignol, 2011), there may be additional vulnerability to excitotoxicity. A recent review by Lee et al. (2015) reports reduced social withdrawal and stereotyped behavior in ASD patients following NMDAr agonist administration suggesting deficits in NMDAr function in ASD. In rodents, down-regulation of NMDArs was related to more social deficits, less communication, and more stereotyped behavior (Gandal et al., 2012). Sensory hyper-reactivity through increased excitation in the auditory, visual and tactile domains has also been reported in ASD (Doyle-Thomas et al., 2014). Furthermore, the down-regulation of GABA, thus reduced inhibition, has been associated with gross and fine motor stereotypes in ASD children such as repetitive simple motor patterns (Gaetz et al., 2014), which suggests a hyper-glutamatergia. Intriguingly, NMDAr antagonist administration has been shown to increase negative symptoms of schizophrenia (Olney et al., 1999; Merritt et al., 2013; Marsman et al., 2014), which are closely related to the social and communication phenotypes of ASD. NMDAr aberrance is therefore thought to lead to symptoms across psychopathology (in Olney et al., 1999).

GABA-glutamate regulation, particularly through NMDAr function, in the auditory cortices has also been associated with aberrant neurophysiological potentials, such as short mismatch negativity latency (Kujala et al., 2007; Kompus et al., 2015), N1 amplitude and γ-band response (Rossignol, 2011; Gandal et al., 2012; McFadden et al., 2012). Such abnormalities have also been identified in schizophrenia (Kirihara et al., 2012; Kärgel et al., 2014) and may lead to potential brain chemical relationship between neuropsychiatric disorder phenotypes (Gandal et al., 2012).

NMDAr antagonism has also led to an increase in glutamine and reduction in glutamate, which might indicate a glutaminase deficiency (Marsman et al., 2013). This increase may also be explained by an increase in glutamine synthetase as a result of reduced nitric oxide production that follows calcium release into the cell due to NMDAr activation Kosenko et al. (2003). NMDAr antagonist administration has been shown to reduce ASD symptoms such as social and cognitive dysfunction, and stereotyped behaviors, suggesting increased functionality or number of NMDArs in ASD (Lee et al., 2015). Decreased glutamate has also been related to increasing negative symptoms in schizophrenia patients (Marsman et al., 2014). Altogether, the literature suggests abnormality in glutamate receptor function that compromises the synthesis of GABA for inhibitory neurotransmission (in Olney et al., 1999; Gandal et al., 2012; Marsman et al., 2013).

Following neuronal excitation by transcranial direct current stimulation (tDCS), ^1^H-MRS measured GABA was reduced, while Glx and isolated glutamine level did not change, indicating no neuronal excitation related change in glutamate level (Stagg et al., 2009). Stagg et al. (2009) infer GABA reduction due to reduced GAD67 activity in response to increased excitatory neuronal firing (Stagg et al., 2009). ^1^H-MRS quantifies only tissue and cyclic glutamate concentration, not NMDAr modulation of glutamate, which may explain the lack of difference in glutamate following neuronal excitation (Stagg et al., 2009). Interestingly, following induced inhibition, both glutamate and GABA concentrations were reduced, suggesting a modulatory effect of glutamate on GAD67 activity (Stagg et al., 2009). This inference counters the hyper-glutamatergic hypothesis that a GAD67 deficiency leads to excess glutamate (Fatemi, 2008). The current review reports decreased GABA and regionally specific reduction in Glx, which, suggests a regulatory role of glutamate in GAD67 production of GABA (Stagg et al., 2009). Concurrently, increased regional glutamate suggests regionally specific hyper-glutamatergia (Fatemi, 2008). Altogether, there is sound evidence for an excitation-inhibition disturbance, specifically hyper-glutmatergia, in ASD that manifests as phenotypes that exist in ASD such as poor social and communication skills (Horder et al., 2013; Doyle-Thomas et al., 2014; Tebartz van Elst et al., 2014).

### 3.4. Glutathione

Glutathione is a protective factor against mitochondrial oxidative stress caused by reactive oxygen species, thus deficiencies lead to the breakdown of mitochondrial function (Rossignol and Frye, 2012; Rae, 2014). Glutathione is generally more abundant in astrocytes, with levels higher in gray matter than white matter (Govindaraju et al., 1998; Rae, 2014) and deficiencies are linked to reduced NAA level, which is essential for neuronal integrity (Govindaraju et al., 1998; Rae, 2014).

Glutathione is extremely difficult to isolate with ^1^H-MRS due to its frequency overlap with glutamate, glutamine, GABA, Cr+PCr, aspartate, and NAA (Govindaraju et al., 1998), even at high magnetic field strengths such as 14T. Radio frequency pulse sequences such as MEGA-PRESS and short TE (Matsuzawa et al., 2008), as well as specialized fitting software, such as LCModel, have been successful in isolating glutathione (Rae, 2014). Nonetheless, there is limited ^1^H-MRS literature discussing the role of glutathione in psychopathology.

To date, no published ^1^H-MRS studies quantify glutathione in ASD samples, though a review of mitochondrial function in ASD reports reduced glutathione and therefore increased mitochondrial dysfunction and oxidative stress (Rossignol and Frye, 2012). Pharmaceutical interventions targeted at increasing glutathione levels have been found to alleviate ASD symptoms (James et al., 2009; Hardan et al., 2012), supporting a deficiency of glutathione in ASD (Rossignol and Frye, 2012). Negative symptoms of schizophrenia have been associated with reduced glutathione in the posterior medial frontal cortex, however gray matter volume differences were not reported in this study (Matsuzawa et al., 2008). Due to the apparent phenotypic link between autism and schizophrenia spectrum disorders (Ford and Crewther, 2014), glutathione level may be related to social cognitive dysfunction in ASD, but clearly more research must be done.

## 4. N-acetylaspartate (NAA)

NAA is predominately synthesized in the mitochondria of neurons, and in oligodendrocytes (Kousi et al., 2013; Rae, 2014). Figure 2 illustrates that aspartate, a product of the Krebs cycle, is synthesized with acetyl coenzyme A (A-CoA), a product of glycolysis, via the enzyme L-aspartate N-acetyltransferase (Asp-Nat) inside the mitochondria, resulting in NAA (Patel and Clark, 1979; Moffett et al., 2007). NAA is then transported to oligodendrocytes, or binds with glutamic acid within the neuronal cytoplasm producing the neurotransmitter NAAG (Kousi et al., 2013; Rae, 2014). In the presynaptic terminal, NAAG activates metabotropic glutamate receptors, releasing NAAG into the synapse that is taken up as NAA by post-synaptic cells, with excess taken up by astrocytes of the blood-brain barrier (Moffett et al., 2007). NAA is catabolised in glial cells and catabolism in oligodendrocytes is a precursor to fatty acids that form the myelin surrounding neuronal axons (Rae, 2014). NAA clearly relies on the integrity of glutamate, Cr+PCr and several other neurochemical processes (Figures 2, 3).

NAA level quantified with ^1^H-MRS is thought to reflect neural density and viability, and indicate neuronal integrity and metabolism (Pouwels and Frahm, 1998; Chugani et al., 1999; Levitt et al., 2003; Kleinhans et al., 2007; Kousi et al., 2013). However, cross-disciplinary studies have challenged this (see Rae, 2014, for a detailed review). NAA has a chemical shift of 2.01 ppm (Kousi et al., 2013; Rae, 2014) with a cortical concentration of NAA from 7.9 to 16.6 mmol/kg_ww_ (Govindaraju et al., 2000). NAAG contributes a amount of it 0.6–2.7 mmol/kg_ww_ (Govindaraju et al., 2000) to the NAA signal due to its close resonant frequency (Kousi et al., 2013; Rae, 2014). NAA is generally comparable across gray and white matter, but is more concentrated in occipital gray matter (Pouwels and Frahm, 1998). NAAG, due to its role in the neurotransmission of NAA, is more abundant in white matter than gray matter (Pouwels and Frahm, 1998).

Table 6 reveals consistently reduced NAA in children with ASD, but not adults, supporting the findings of the meta-analysis by Aoki et al. (2012). Widespread reduction in NAA and NAA/Cr+PCr suggests dysfunction, loss or immaturity of neurons (DeVito et al., 2007; Gabis et al., 2008; Aoki et al., 2012; Horder et al., 2013), particularly in the mitochondria (Endo et al., 2007), and reduced axon density (Levitt et al., 2003). Therefore, according to Aoki et al. (2012), the hallmark cortical density of ASD might be caused by non-neuronal factors such as excess glial cells and myelination, enlarged glial cells, and/or premature myelination. Across childhood, patterns of gray and white matter NAA level differ between ASD and typically developing children. In typically developing 3–10 year olds, there is overall gradual increase in gray matter NAA. In contrast, children with ASD have a high NAA level early in development, which plateaus from 6–7 years, and then continues to increase to abnormaly high levels by 9–10 years of age. A similar trajectory is seen in white matter, although for typically developing children there is a peak at 6–7 years (Corrigan et al., 2013). Earlier studies support these findings (Zeegers et al., 2007; O'Brien et al., 2010). NAA levels seem to plateau in adulthood ASD, but continues to increase in controls reflecting compromised neural density and integrity throughout the lifespan (O'Brien et al., 2010), further highlighting an aberrant NAA trajectory in ASD.

**Table 6:** **^1^H-MRS quantified NAA differences for children and adults with ASD**.

**Region**				**Children**				**Adult**		
				**NAA**		**NAA/Cr+PCr**		**NAA**		**NAA/Cr+PCr**
GM			⇊	Corrigan et al., 2013^†^						
				Corrigan et al., 2013^‡^^#^						
			⇊	Friedman et al., 2006						
	Frontal		⇊	DeVito et al., 2007						
	Occipital		⇊	DeVito et al., 2007						
	Temporal		↓	DeVito et al., 2007						
WM			⇊	Corrigan et al., 2013^†^^#^						
				Corrigan et al., 2013^‡^						
			⇊	Friedman et al., 2006						
			↓	DeVito et al., 2007						
	Frontal			Zeegers et al., 2007						
		Left	↓	Friedman et al., 2003						
			⇊	Levitt et al., 2003						
		Right		Friedman et al., 2003						
				Levitt et al., 2003						
	Parietal	Left	⇊	Friedman et al., 2003						
			⇊	Levitt et al., 2003						
		Right	↓	Friedman et al., 2003						
				Levitt et al., 2003						
Frontal				Hisaoka et al., 2001	⇊	Kubas et al., 2012				
				Levitt et al., 2003						
		Left		Harada et al., 2011		Vasconcelos et al., 2008	⇊	Kleinhans et al., 2007		
		Right		Chugani et al., 1999						
	MPF	Right				Endo et al., 2007	↑	Murphy et al., 2002		Murphy et al., 2002
	DLPFC	Left			⇊	Fujii et al., 2010	⇊	Horder et al., 2013		
		Right				Fujii et al., 2010				Oner et al., 2007
Parietal				Hisaoka et al., 2001						
				Levitt et al., 2003						
		Left						Horder et al., 2013		
								Kleinhans et al., 2007		
		Right				Hashimoto et al., 1997		Page et al., 2006		Page et al., 2006
						Hashimoto et al., 1998		Murphy et al., 2002		Murphy et al., 2002
IPS		Left						Bernardi et al., 2011		
		Right					↓	Bernardi et al., 2011		
Temporal			⇊	Hisaoka et al., 2001						
		Left		Chugani et al., 1999						
	MTL			Friedman et al., 2003						
		Right			⇊	Endo et al., 2007				
Auditory							↑	Brown et al., 2013		
TPJ		Left						Bernardi et al., 2011		
		Right					↓	Bernardi et al., 2011		
STG		Left		Friedman et al., 2003						
		Right	⇊	Friedman et al., 2003						
Occipital				Friedman et al., 2003				Kleinhans et al., 2007		
				Levitt et al., 2003						
HAC					⇊	Gabis et al., 2008		Kleinhans et al., 2009		Kleinhans et al., 2009
		Left		Zeegers et al., 2007				Suzuki et al., 2010		
		Right	⇈	O'O'Brien et al., 2010	⇈	O'Brien et al., 2010		Page et al., 2006		Page et al., 2006
			⇊	Otsuka et al., 1999				O'Brien et al., 2010		O'Brien et al., 2010
Thalamus			⇊	Hardan et al., 2008		Doyle-Thomas et al., 2014		Bernardi et al., 2011		
			⇊	Friedman et al., 2003						
				Levitt et al., 2003						
Cerebellum			↓	DeVito et al., 2007		Gabis et al., 2008				
		Left	⇊	Chugani et al., 1999		Vasconcelos et al., 2008		Tebartz van Elst et al., 2014		
			⇊	Otsuka et al., 1999						
		Right					⇊	Suzuki et al., 2010		
								Kleinhans et al., 2007		
Cingulate				Hisaoka et al., 2001						
		Left	⇊	Friedman et al., 2003						
		Right	↓	Friedman et al., 2003						
Putamen				Levitt et al., 2003						
		Left	⇊	Friedman et al., 2003						
		Right		Friedman et al., 2003						
ACC				Levitt et al., 2003	⇊	Fujii et al., 2010	⇊	Tebartz van Elst et al., 2014		
						Vasconcelos et al., 2008		Bernardi et al., 2011		
		Left		Bejjani et al., 2012						
		Right	⇈	Bejjani et al., 2012					↑	Oner et al., 2007
Caudate				Friedman et al., 2003						
*Head*				Levitt et al., 2003		Doyle-Thomas et al., 2014				
*Body*		Left	⇊	Levitt et al., 2003						
		Right		Levitt et al., 2003						
Lent		Left		Harada et al., 2011						
Callosum				Friedman et al., 2003						
Insula				Friedman et al., 2003						
Vermis						Endo et al., 2007		Kleinhans et al., 2007		
Centsemi		Left				Fayed and Modrego, 2005				
Striatum		Left				Vasconcelos et al., 2008				
Brainstem				Hisaoka et al., 2001						
BasGan		Left					⇊	Horder et al., 2013		

1.5T, 3T; ⇊, p < 0.001; ↓, p <, 0.01; ⇈, p < 0.001; ↑, p < 0.01;

† 3–4 years;

‡ 6–7 years;

# 9–10 years;

*ACC, Anterior Cingulate Cortex; BasGan, Basal Ganglia; Cent Semi, Centrum Semiovale; Cr, Creatine+Phosphocreatine; DLPFC, Dorsolateral Prefrontal Cortex; GM, Gray Matter; HAC, Hippocampus-Amygdala Complex; Intraparietal Suclus; Lent, Lenticular Nuclei; MPF, Medial Prefrontal; MPL, Medial Parietal Lobe; MTL, Medial Temporal Lobe; STG, Superior Temporal Gyrus; TPJ, Temporo-parietal Junction; WM, White Matter*.

In children with ASD, reduced parietal axon density, marked by reduced white matter NAA, is associated with deficits in socially directed eye gaze, spatial perception, and memory (Levitt et al., 2003). Similarly, reduced NAA/Cr+PCr level in the ACC is associated with poorer social functioning (Fujii et al., 2010), and in the right medial temporal lobe (MTL) with poor emotional and listening response (Endo et al., 2007). Furthermore, NAA deficits in Wernicke's language center on the left, and auditory interpretation, non-verbal communication and memory on the right may associated with language deficits (Hisaoka et al., 2001) and NAA/Cr+PCr deficit in regions responsible for executive functions may explain social and communication disabilities (Fujii et al., 2010; Horder et al., 2013).

In adults with ASD, deficits in social responsiveness worsen with higher levels of auditory (Brown et al., 2013) and prefrontal cortex NAA (Murphy et al., 2002). NAA deficit in the HAC have been suggested to cause communication difficulties through compromised integrity of neurons in the amygdala during development rather than adulthood (Kleinhans et al., 2009). There was no relationship with social avoidance and distress. The HAC is thought to be central to ASD related behaviors (Gabis et al., 2008), with reduced NAA in the HAC associated with repetitive behaviors in adults with ASD (Kleinhans et al., 2009), while increased ACC and decreased DLPFC NAA/choline was related to more obsessive compulsive behaviors (Oner et al., 2007). The temporal lobes and limbic system, which include the HAC and ACC, are in close proximity and connectivity. Considering this networks involvement in emotion processing, motor response to emotional cues and attention, it is of little surprise that marked social and emotional difficulties may manifest from an NAA deficiency.

Reduced frontal NAA level has been identified in schizophrenia studies (Marsman et al., 2013), suggesting that common neuronal impairment and/or loss (Kleinhans et al., 2007; Horder et al., 2013; Marsman et al., 2014) may explain similarities in social and cognitive deficits between the disorders. Altogether, NAA level abnormalities may underpin some of the central phenotypes of ASD: verbal and non-verbal social communication and interaction. However, inconsistencies across methodologies and assessment of ASD and control groups, and the interconnectedness of metabolic pathways, such as the synthesis of NAAG, must be considered when quantifying NAA with ^1^H-MRS.

## 5. Choline containing compounds

Choline containing compounds are essential components of cellular membranes, and necessary for the synthesis of the neurotransmitter acetylcholine (ACh). Choline is typically synthesized in the liver and is transported across the blood-brain barrier, as it cannot be synthesized in the brain *de novo*. Choline typically crosses into the brain in the form of phosphatidylcholine or lysophosphatidylcholine (Rae, 2014). Once in the brain, several enzymic reactions take place to synthesize choline containing compounds and ACh; see Figure 2 for an illustration, and Rae (2014) for a detailed description.

In ^1^H-MRS, the choline peak contains both phosphorylcholine (PCh) and glycerophosphorylcholine (GPCh), with a small contribution of ACh and free choline (Govindaraju et al., 2000; Rae, 2014), referred to hereafter as choline+. Choline+ resonates at 3.2 ppm (Govindaraju et al., 1998; Kousi et al., 2013), with concentration varying between 0.9 and 2.5 mmol/kg_ww_ across the brain (Govindaraju et al., 2000). Choline moieties are highly interconnected, and are at equilibrium with membrane phospholipids that make up 40% of myelin. Due to its role in myelination, ^1^H-MRS measured levels of choline+ are largest in white matter (Pouwels and Frahm, 1998; Bertholdo et al., 2013). Thus, measured cortical choline+ levels indicate cellular membrane metabolism (Pouwels and Frahm, 1998), specifically the equilibrium of membrane phospholipid anabolism and catabolism (Pouwels and Frahm, 1998; Blüml et al., 1999; Gabis et al., 2008; Suzuki et al., 2010; Bertholdo et al., 2013; Rae, 2014), with elevated levels in childhood thought to be due to membrane phospholipid anabolism for myelin growth (Blüml et al., 1999). The cholinergic system also plays a role in cognitive development and function (Lam et al., 2006).

Choline+ levels in children are generally reduced, particularly in cortical gray matter, temporal regions and the left thalamus, suggesting a decrease in membrane phospholipid turnover (Table 7; Friedman et al., 2003, 2006; Levitt et al., 2003; Fayed and Modrego, 2005; DeVito et al., 2007; Hardan et al., 2008; Corrigan et al., 2013). These data are also indicative of neurodevelopmental delay as a result of reduced glial cell density (in Baruth et al., 2013) and deficient myelination leading to slower neural processes (Corrigan et al., 2013). By contrast, increases in choline+ in the caudate (Levitt et al., 2003), and choline+/Cr+PCr in the ACC (Vasconcelos et al., 2008) and HAC (Gabis et al., 2008) of children with ASD marks regionally abnormal membrane phospholipid turnover (Vasconcelos et al., 2008). Choline+/Cr+PCr in the HAC progressively decreased from 10–50 years of age in AS, but not controls (O'Brien et al., 2010), contradicting the broader findings of choline+ that is reduced in children, but increased in adults, with ASD (Table 7). Nonetheless, findings suggest differences in neural membrane maturation between control and ASD groups (O'Brien et al., 2010).

**Table 7:** **^1^H-MRS quantified total choline (Cho) differences for children and adults with ASD**.

**Region**				**Children**				**Adult**		
				**Choline**		**Cho/Cr+PCr**		**Choline**		**Cho/Cr+PCr**
GM			⇊	Corrigan et al., 2013^†^^‡^						
				Corrigan et al., 2013^#^						
			⇊	Friedman et al., 2006						
			⇊	Fayed and Modrego, 2005						
	Frontal		↓	DeVito et al., 2007						
	Occipital			DeVito et al., 2007						
	Temporal			DeVito et al., 2007						
WM			⇊	Corrigan et al., 2013^†^						
				Corrigan et al., 2013^‡^^#^						
				Friedman et al., 2006						
				Fayed and Modrego, 2005						
				DeVito et al., 2007						
	Frontal			Friedman et al., 2003						
				Levitt et al., 2003						
				Zeegers et al., 2007						
	Parietal			Friedman et al., 2003						
				Levitt et al., 2003						
Frontal				Hisaoka et al., 2001		Kubas et al., 2012				
				Levitt et al., 2003						
		Left				Vasconcelos et al., 2008				
	MPF	Right				Endo et al., 2007	⇈	Murphy et al., 2002		
	DLPFC	Left				Fujii et al., 2010		Horder et al., 2013		
		Right				Fujii et al., 2010				Oner et al., 2007
Parietal				Levitt et al., 2003						
				Hisaoka et al., 2001						
		Left						Horder et al., 2013		
		Right				Hashimoto et al., 1997		Page et al., 2006		Page et al., 2006
						Hashimoto et al., 1998				
	MPL	Right						Murphy et al., 2002		
IPS								Bernardi et al., 2011		
Temporal				Hisaoka et al., 2001						
	MTL		⇊	Friedman et al., 2003		Endo et al., 2007				
Auditory								Brown et al., 2013		
TPJ								Bernardi et al., 2011		
STG		Left		Friedman et al., 2003						
		Right	⇊	Friedman et al., 2003						
Occipital				Friedman et al., 2003						
				Levitt et al., 2003						
HAC								Kleinhans et al., 2009		Kleinhans et al., 2009
		Left		Zeegers et al., 2007	⇈	Gabis et al., 2008	⇈	Suzuki et al., 2010		
		Right		Otsuka et al., 1999		Gabis et al., 2008				
				O'Brien et al., 2010		O'Brien et al., 2010		Page et al., 2006		Page et al., 2006
								O'Brien et al., 2010		O'Brien et al., 2010
Thalamus				Levitt et al., 2003		Doyle-Thomas et al., 2014		Bernardi et al., 2011		
		Left	⇊	Friedman et al., 2003						
			⇊	Hardan et al., 2008						
		Right		Friedman et al., 2003						
				Hardan et al., 2008						
Cerebellum				DeVito et al., 2007	⇈	Gabis et al., 2008				
		Left		Otsuka et al., 1999		Vasconcelos et al., 2008		Tebartz van Elst et al., 2014		
		Right						Suzuki et al., 2010		
Cingulate				Hisaoka et al., 2001						
				Friedman et al., 2003						
Putamen				Levitt et al., 2003		Doyle-Thomas et al., 2014				
				Friedman et al., 2003						
ACC				Bejjani et al., 2012		Fujii et al., 2010		Tebartz van Elst et al., 2014		
		Left			⇈	Vasconcelos et al., 2008		Bernardi et al., 2011		
	Inferior		⇊	Levitt et al., 2003						
	Superior			Levitt et al., 2003						
		Right								Oner et al., 2007
	Inferior			Levitt et al., 2003						
	Superior			Levitt et al., 2003						
Caudate				Friedman et al., 2003		Doyle-Thomas et al., 2014				
*Head*		Left		Levitt et al., 2003						
		Right	⇈	Levitt et al., 2003						
*Body*				Levitt et al., 2003						
BasGan		Left					⇊	Horder et al., 2013		
Callosum				Friedman et al., 2003						
Insula				Friedman et al., 2003						
Vermis						Endo et al., 2007				
Centsemi		Left				Fayed and Modrego, 2005				
Striatum		Left				Vasconcelos et al., 2008				
Brainstem				Hisaoka et al., 2001						

1.5T, 3T; ⇊, p < 0.001; ↓, p < 0.01; ⇈, p < 0.001; ↑, p < 0.01;

† 3–4 years;

‡ 6–7 years;

# 9–10 years;

*ACC, Anterior Cingulate Cortex; Ant, Anterior; BasGan, Basal Ganglia; Cent Semi, Centrum Semiovale; Cr+PCr, Creatine+Phosphocreatine; DLPFC, Dorsolateral Prefrontal Cortex; GM, Gray Matter; HAC, Hippocampus-Amygdala Complex; IPS, Intraparietal Suclus; MPF, Medial Prefrontal; MPL, Medial Parietal Lobe; MTL, Medial Temporal Lobe; Post, Posterior; STG, Superior Temporal Gyrus; TPJ, Temporo-parietal Junction; WM, White Matter*.

Alternatively, in adolescents and adults with ASD high choline+ concentration (Table 7) might indicate the catabolism of membrane phospholipids, or “active demyelination” (Murphy et al., 2002; Gabis et al., 2008; Suzuki et al., 2010; Bertholdo et al., 2013). However, as choline+ marks both synthesis and degradation of membrane phospholipids, high choline+ levels may also be an indication of increased cellular proliferation and density, thus increased synthesis and metabolism (Murphy et al., 2002; Sokol et al., 2002; Suzuki et al., 2010); for a review see Baruth et al. (2013).

Thalamic choline+/Cr+PCr ratio has been shown to decrease with increasing severity of communication deficits, and restricted and repetitive behaviors in children with ASD (Doyle-Thomas et al., 2014). In adults with ASD on the other hand, prefrontal and HAC choline+ increases with communication deficits (Murphy et al., 2002) and aggression(Suzuki et al., 2010). These data suggest that communication deficits have different regional and functional origins. Somewhat contradictory to the general findings in children, is that MTL choline+ (Endo et al., 2007) and HAC choline+/Cr+PCr (Sokol et al., 2002) increase with severity of autistic symptoms, although medication (Sokol et al., 2002) and Cr/PCr levels may be a mediating factor. For adults, there was no relationship between symptom severity and choline+ level (Page et al., 2006; Kleinhans et al., 2009).

In sum, choline+ reduction in children with ASD, and increase during adulthood, indicates widespread and lasting abnormalities in cellular and myelin integrity. Cholinergic pathways have been linked to social and behavioral abnormalities, particularly in ASD symptom severity (Lam et al., 2006), and orientation of attention and sensory processing through electrophysiological change detection studies (Orekhova and Stroganova, 2014), calling for further research into behavioral correlates of choline containing compounds.

## 6. Myo-inositol

myo-Inositol, a form of the simple sugar-alcohol Inositol, is synthesized predominantly in the kidney, with a small proportion ingested, entering the brain via a plasma membrane myo-Inositol transporter. Some myo-Inositol is also synthesized in the cytoplasm from glucose via glycolysis, as illustrated in Figure 2 (Ross and Bluml, 2001; Rae, 2014). myo-Inositol is a component of lipid biomembranes through phosphoglycerides (phosphatidylinositol and phosphatidylinositol phosphate), and is involved in the regulation of brain cell volume as an organic osmolyte (Ross and Bluml, 2001; Rae, 2014). myo-Inositol is thought to be a marker of astrocytes (Pouwels and Frahm, 1998; Ross and Bluml, 2001; Kousi et al., 2013), and plays an important role in the maintenance of metabolism (Ross and Bluml, 2001), and in brain cell signaling as an intracellular post-receptor second messenger system. This second messenger system is linked to several receptors (including glutamate receptors) in the central nervous system (Rae, 2014). myo-Inositol resonates at 3.56ppm in ^1^H-MRS (Pouwels and Frahm, 1998; Ross and Bluml, 2001; Kousi et al., 2013) with a concentration of between 3.1 and 8.1 mmol/kg_ww_ (Govindaraju et al., 2000), and tends to be greater in gray than white matter. This is contradictory to speculation that myo-Inositol is an index of myelin breakdown (Ross and Bluml, 2001; Rae, 2014).

Table 8 illustrates reduced myo-Inositol concentration across the childhood ASD brain. On the other hand, myo-Inositol/Cr+PCr ratio in some regions is increased, which might be due to the mediation of Cr+PCr (Gabis et al., 2008; Rae, 2014). It should also be noted, that regional differences were measured at 1.5T and no differences were found at 3T, which may reflect a limitation in scanner strength, and that standard scanning protocols are suboptimal for the quantification of myo-Inositol as a short T2 relaxation time requires a short TE for peak isolation (Bertholdo et al., 2013; Kousi et al., 2013). Reduced myo-inositol in adults with ASD is only reported in the temporo-parietal junction at 3T (Bernardi et al., 2011). myo-Inositol deficits indicate reduced glial cell proliferation and brain signaling, while the converse is implied by increased myo-Inositol. Altogether, regionally specific reduced myo-Inositol in children with ASD suggests reduction in cell signaling and/or volume regulation in those regions.

**Table 8:** **^1^H-MRS quantified myo-Inositol (mI) differences for children and adults with ASD**.

**Region**				**Children**				**Adult**		
				**myo-Inositol**		**mI/Cr**		**myo-Inositol**		**mI/Cr+PCr**
GM				Corrigan et al., 2013^†^^‡^^#^						
			⇊	Friedman et al., 2006						
	Frontal			DeVito et al., 2007						
	Occipital			DeVito et al., 2007						
	Temporal			DeVito et al., 2007						
WM				Corrigan et al., 2013^†^^‡^^#^						
			↓	Friedman et al., 2006						
				DeVito et al., 2007						
	Frontal	Left		Friedman et al., 2003						
		Right	↓	Friedman et al., 2003						
	Parietal	Left	⇊	Friedman et al., 2003						
		Right		Friedman et al., 2003						
Frontal						Kubas et al., 2012				
		Left				Vasconcelos et al., 2008				
	DLPFC	Left						Horder et al., 2013		
Parietal		Left						Horder et al., 2013		
		Right						Page et al., 2006		Page et al., 2006
IPS								Bernardi et al., 2011		
Temporal	MTL			Friedman et al., 2003						
Auditory								Brown et al., 2013		
TPJ							⇊	Bernardi et al., 2011		
STG				Friedman et al., 2003						
Occipital			⇊	Friedman et al., 2003						
HAC					⇈	Gabis et al., 2008		Kleinhans et al., 2009		Kleinhans et al., 2009
		Right		O'Brien et al., 2010				O'Brien et al., 2010		
								Page et al., 2006		Page et al., 2006
Thalamus				Friedman et al., 2003		Doyle-Thomas et al., 2014		Bernardi et al., 2011		
				Hardan et al., 2008						
Cerebellum				DeVito et al., 2007	⇈	Gabis et al., 2008				
		Left				Vasconcelos et al., 2008		Tebartz van Elst et al., 2014		
Cingulate				Friedman et al., 2003						
Putamen				Friedman et al., 2003		Doyle-Thomas et al., 2014				
ACC				Bejjani et al., 2012	⇈	Vasconcelos et al., 2008		Tebartz van Elst et al., 2014		
								Bernardi et al., 2011		
Caudate						Doyle-Thomas et al., 2014				
			⇊	Friedman et al., 2003						
Callosum*Anterior*			⇊	Friedman et al., 2003						
*Post*				Friedman et al., 2003						
Insula		Left	↓	Friedman et al., 2003						
		Right	⇊	Friedman et al., 2003						
Centsemi		Left				Fayed and Modrego, 2005				
Striatum		Left			⇈	Vasconcelos et al., 2008				
BasGan		Left						Horder et al., 2013		

1.5T, 3T; ⇊, p < 0.001; ↓, p < 0.01; ⇈, p < 0.001; ↑, p < 0.01;

† 3–4 years;

‡ 6–7 years;

# 9–10 years;

*ACC, Anterior Cingulate Cortex; Ant, Anterior; BasGan, Basal Ganglia; Cent Semi, Centrum Semiovale; Cr+PCr, Creatine+Phosphocreatine; DLPFC, Dorsolateral Prefrontal Cortex; GM, Gray Matter; HAC, Hippocampus-Amygdala Complex; IPS, Intraparietal Suclus; MTL, Medial Temporal Lobe; Post, Posterior; STG, Superior Temporal Gyrus; TPJ, Temporo-parietal Junction; WM, White Matter*.

## 7. Lactate

Lactate is synthesized via the enzyme lactate dehydrogenase from pyruvate, which is synthesized from glucose from the capillary, in both astrocytes and neurons, as illustrated in Figures 2, 3. Lactate is then transported back to the capillaries via mono-carboxylate transporters (Rae, 2014). Lactate is integral to many cortical cellular metabolic processes and pathways, as well as signaling, acting as a shuttle between and within cells that deliver oxidative and gluconeogenic substrates (Gladden, 2004; Brooks, 2009). In ^1^H-MRS, lactate resonates at 1.33 ppm, however as a major energy source for neurons the concentration is low at rest and is contaminated by lipids and MMs making quantification difficult (Friedman et al., 2006; Kousi et al., 2013; Rae, 2014). ^1^H-MRS protocols with a long TE sequence can be used to optimize lactate quantification (Friedman et al., 2003, 2006; Corrigan et al., 2013).

ASD studies have reported lactate abnormalities throughout the body; for a comprehensive review see Rossignol and Frye (2012). However very few ASD ^1^H-MRS studies report cortical concentrations of lactate and of these, quantification is attempted using a 1.5T scanner, which is inadequate for lactate quantification thus should be interpreted with caution (Bertholdo et al., 2013; Kousi et al., 2013). As such, no differences have been reported across the cortex (Hashimoto et al., 1997; Chugani et al., 1999; Friedman et al., 2003, 2006; Corrigan et al., 2013). One study of 15 ASD and 15 controls reported a lactate signal in only one child with ASD (Chugani et al., 1999). To date, no adult ASD ^1^H-MRS studies investigate lactate.

## 8. Limitations

### 8.1. ^1^H-MRS research

Although several metabolic processes appear to differ between ASD and control groups, Table 2 illustrates several methodological differences between studies. Differences in scanner strength, pulse sequence protocols, water suppression techniques, the use of a “stable” metabolite reference, and variable regions of interest contribute to inconsistent findings, and will be discussed in turn.

Of the research presented in this review, 80% employ a 1.5T MR scanner which has suboptimal spatial and spectral resolution (Bertholdo et al., 2013), SNR (Kousi et al., 2013; Juchem and Rothman, 2014) and more statistical variation (Marsman et al., 2013) compared to those at 3T and above. 1.5T is also insufficient to distinguish glutamate, glutamine, and GABA (Page et al., 2006; Aoki et al., 2012; Juchem and Rothman, 2014); it is of concern that two studies report isolated glutamate at 1.5T (Hardan et al., 2008; Hassan et al., 2013). MMs are also a concern in the quantification of glutamate, glutamine and GABA resonance due to their invisibility in NMR (Ross and Bluml, 2001). To combat these limitations, variations in TE can be used to optimize metabolite isolation. A shorter TEs (30–40 ms) provides a larger signal, and more clearly resolve myo-Inositol, glutamate, glutamine and lipids (Agzarian and Walls, 2011; Bertholdo et al., 2013). A long TE (greater than 54 ms) and spectral editing techniques (e.g., MEGA-PRESS) are thought to be effective in suppressing MM's from the spectra (Agzarian and Walls, 2011), and an 80 ms TE has been shown to resolve glutamate and glutamine (Schubert et al., 2004). Long TEs (135–288 ms) have also been shown to reduce baseline noise (Agzarian and Walls, 2011). These techniques are not as effective with GABA isolation and quantification, so MMs are a known limitation of GABA quantification (Ganji et al., 2012; Mullins et al., 2014).

Cortical water masks metabolite peaks and therefore must be suppressed to expose metabolite levels (Kousi et al., 2013). Suppression is achieved by frequency-selective pulse-sequences such as chemical shift selective water suppression (CHESS) and relaxation time manipulations (Bertholdo et al., 2013; Kousi et al., 2013; Juchem and Rothman, 2014). ^1^H-MRS statistical analysis software, such as LCModel, can also account for cortical water variation (Provencher, 2001). Insufficient and variable water suppression methods therefore contribute to variation in quantified metabolite levels across studies.

Forty percent of ASD ^1^H-MRS studies report metabolite levels as a ratio to a reference signal, such as Cr+PCr, which are subject to variation between experimental groups (Levitt et al., 2003; Harada et al., 2011; Rojas et al., 2011; Doyle-Thomas et al., 2014). In fact, Endo et al. (2007) suggest that their finding of low NAA/Cr+PCr may be a reflection of high Cr+PCr. These inconsistencies across studies impact the interpretability of the literature at large and should be taken into account.

Finally, region of interest varies significantly across studies (Sokol et al., 2002; Harada et al., 2011) and small sample size leads to low statistical power(Bernardi et al., 2011) that affects generalizability. Although there are benefits in reporting metabolite levels across a the cortex, it is difficult to draw inferences regarding their regionally specific role due to the lack of study replications. Furthermore, within regions of interest is a variation in tissue composition that affects the overall metabolite level. For example, white and gray matter contains substantially different amounts of neurons and oligodendrocytes, and white matter astrocytes differ to those in gray matter (Amaral et al., 2013). The level of Cr+PCr, glutamate, glutamine, NAA and myo-Inositol is higher in gray matter, which comprise cell bodies and glia. Choline+ level is highest in white matter, comprising mostly axons and glia (Pouwels and Frahm, 1998). Furthermore, energy consumption also differs between tissues with gray matter generally requiring more energy than white matter (Amaral et al., 2013).

Altogether, it is clear that ^1^H-MRS research should take into account the variation in methodologies when developing studies and making inferences regarding the literature at large. Efforts should be made to replicate methods and cortical regions of interest to establish generalisability. This is essential for the use of ^1^H-MRS to inform the theoretical framework of metabolic behavior within the healthy and diseased brain.

### 8.2. ASD research

In addition to ^1^H-MRS methodology inconsistencies, experimental groups themselves vary in diagnosis, age, intelligence, trait behaviors and phenotypes, medications, and family history as illustrated in Table 1. These are discussed in turn below.

In terms of the ASD diagnosis itself, research suggests that the triad of phenotypes is genetically heterogeneous (Happé et al., 2006; Ronald et al., 2006; Robinson et al., 2012). Furthermore, certain metabolites have been related to specific symptoms, such as communication and empathy with glutamate (Horder et al., 2013; Tebartz van Elst et al., 2014), communication and restricted and repetitive behaviors with choline+ (Doyle-Thomas et al., 2014), and social responsiveness with NAA(Murphy et al., 2002; Brown et al., 2013). These symptoms manifest as trait phenotypes in non-clinical control groups (Baron-Cohen et al., 2001). However, information regarding ASD trait phenotypes within control groups is seldom reported, questioning the integrity of group comparisons and the relationship between metabolite levels and phenotypes. Thus, it is possible that the largely inconsistent results reported in this review are a product of non-specific group membership (Kleinhans et al., 2007). Furthermore, ASD phenotypes are comorbid with psychosis (Stahlberg et al., 2004; Bakken et al., 2007; Solomon et al., 2011), schizophrenia (Stahlberg et al., 2004; Rapoport et al., 2009; Solomon et al., 2011), schizoid personality disorder (Coolidge et al., 2013; Dinsdale et al., 2013; Ford and Crewther, 2014), schizotypal traits (Dinsdale et al., 2013; Ford and Crewther, 2014), epilepsy and mental retardation (Aoki et al., 2012), ADHD (Stahlberg et al., 2004; Brieber et al., 2007), bipolar disorder (Stahlberg et al., 2004), and anxiety (Stahlberg et al., 2004). It is therefore important that ASD and other multi-dimensional neurological disorder research focus on biological markers of specific symptom phenotypes, rather than psychiatric disorders as a whole.

The role of metabolite levels in intelligence remains relatively unknown, although performance IQ has been related to increased right HAC NAA/Cr+PCr and myo-Inositol/Cr+PCr in ASD, while HAC myo-Inositol/Cr+PCr has been inversely related to performance IQ for controls (Gabis et al., 2008). Although many find no relationship between intelligence and metabolites discussed in this review(Page et al., 2006; Hardan et al., 2008; Fujii et al., 2010; Suzuki et al., 2010; Bernardi et al., 2011; Rojas et al., 2011; Brown et al., 2013; Gaetz et al., 2014), Table 1 illustrates that several ^1^H-MRS studies do not match intelligence between experimental groups (Levitt et al., 2003; Endo et al., 2007; Kleinhans et al., 2007; Gabis et al., 2008; Hardan et al., 2008; O'Brien et al., 2010; Rojas et al., 2011; Bejjani et al., 2012; Brown et al., 2013).

Sex differences in metabolite levels are also relatively unknown, although girls are reported to have reduced choline+/Cr+PCr ratio in the thalamus (Doyle-Thomas et al., 2014) and increased choline+ in the right caudate (Levitt et al., 2003). Metabolite level differences across age have been reported extensively (Hisaoka et al., 2001; DeVito et al., 2007; Zeegers et al., 2007; O'Brien et al., 2010; Aoki et al., 2012; Kubas et al., 2012; Corrigan et al., 2013; Doyle-Thomas et al., 2014), particularly the level of frontal NAA (DeVito et al., 2007; Zeegers et al., 2007; Aoki et al., 2012; Kubas et al., 2012) and Cr+PCr (Zeegers et al., 2007), temporal NAA (Hisaoka et al., 2001; DeVito et al., 2007), HAC NAA (O'Brien et al., 2010) and Glx (DeVito et al., 2007; Doyle-Thomas et al., 2014), and motor and visual GABA/Cr+PCr (Gaetz et al., 2014). These studies suggest that NAA, Cr+PCr, choline+ and myo-Inositol abnormalities in childhood may normalize through adolescence to adulthood for those with ASD. Clinical symptoms related to NAA and choline+ may reflect developmental abnormalities rather than state-specific social deficits (Kleinhans et al., 2009). Furthermore, age related changes appear to differ between ASD and typically developing groups (DeVito et al., 2007; Corrigan et al., 2013).

A major implication for studies of this nature concerns the administration of pharmacological interventions, whether for sedation or patient medication. Due to the role of psychotropic medications on neurotransmitter systems such as the serotonergic, dopaminergic and glutamatergic, metabolite levels may be affected (Lam et al., 2006; Joshi et al., 2013).

However these medications often do not account for differences in metabolic processes (Sokol et al., 2002; Vasconcelos et al., 2008; Rojas et al., 2011; Joshi et al., 2013; Gaetz et al., 2014), and no differences between medicated and unmedicated participants are reported (DeVito et al., 2007; Bejjani et al., 2012; Tebartz van Elst et al., 2014). Sample size is often too small to draw meaningful conclusions however Oner et al. (2007) and Sokol et al. (2002) suggest that medications may have an indirect effect in ASD, with symptom severity significantly associated with choline+/Cr+PCr increase for all ASD children, but not unmedicated children alone. Levitt et al. (2003) report lower Cr+PCr level in the right caudate head of medicated vs. unmedicated children, concluding that therapeutic drugs may normalize Cr+PCr. Sedatives were administered in the majority of child ^1^H-MRS studies, particularly to the ASD group. The effect of the sedative triclofos is unclear, although it may promote GABA levels through increased GABA transaminase (Harada et al., 2011). The sedative midazlolam appears to have no effect (18 sedated, eight un-sedated) on NAA and Glx level for children with ASD (DeVito et al., 2007). Control participants are rarely sedated so it cannot be determined whether sedatives affect metabolic processes in typically developing children (DeVito et al., 2007). In sum, the effects of pharmacological interventions should be analyzed and reported, failing this the results should be interpreted with caution.

## 9. Conclusion

Future research should recognize the limitations of the current literature, both in ^1^H-MRS protocols and participant selection. Specifically, it is important that studies record and report ASD phenotypes across all experimental groups. This practice is beneficial two-fold as it ensures the control group is not affecting differences between the groups, and will provide greater insight into the neurochemical role in the broader phenotype of ASD. Collecting trait level data need not be specific to ASD studies however, all research investigating spectrum disorders would benefit from such a practice.

We summarize the extensive and intricate network of metabolic activity specific to ASD symptoms, while highlighting current ASD research shortcomings, namely; inconsistent ^1^H-MRS protocols, limited phenotype data, ASD heterogeneity, and clinical and control samples variability. These ultimately limit the development of ASD theory as it is difficult to draw a meaningful conclusion from the literature at large. Nevertheless, reduced absolute levels of sub-cortical NAA and Cr+PCr, cortical white matter Cr+PCr, Glx, NAA and myo-Inositol, and gray matter Cr+PCr, Glx, NAA, and choline+ are somewhat consistent in children with ASD. Inconsistent results in adult studies however, may reflect the aforementioned limitations. Clinical and trait ASD phenotypes are largely understudied and should be used to identify abnormalities in the metabolic pathway from which pervasive symptoms arise. Ultimately, phenotype-specific studies will advance what is known of the underpinnings of ASD, and the detection, diagnosis and treatment of ASD and other multi-dimensional psychiatric disorders.

## Author contributions

TF conducted the majority of the literature search and writing of the critical review. Professor DC played a key role in the development of the argument and critical analysis of the literature, and establishing the means to best represent the data. Both authors read and approved the final version of the manuscript.

## Funding

The National Health and Medical Research Council of Australia, APP1004740.

### Conflict of interest statement

The authors declare that the research was conducted in the absence of any commercial or financial relationships that could be construed as a potential conflict of interest.
